# A TIR‐NLR gene from Arabidopsis Pla‐1 confers resistance to geminivirus infection

**DOI:** 10.1111/tpj.70628

**Published:** 2025-12-06

**Authors:** Wei Shen, Rajrani Ruhel, Maria Ines Reyes, Emily Wheeler, David Deppong, Leigh Mickelson‐Young, Joseph Ndunguru, Linda Hanley‐Bowdoin, José Trinidad Ascencio‐Ibáñez

**Affiliations:** ^1^ Department of Plant and Microbial Biology North Carolina State University Raleigh North Carolina USA; ^2^ Tanzania Plant Health and Pesticide Authority Arusha Tanzania; ^3^ Department of Molecular and Structural Biochemistry North Carolina State University Raleigh North Carolina USA; ^4^ Present address: Department of Developmental Biology and Center of Regenerative Medicine Washington University School of Medicine in St. Louis St. Louis Missouri USA; ^5^ Present address: Seed Technology and Analytics BASF Corporation Research Triangle Park North Carolina USA

**Keywords:** plant virus, geminivirus, CaLCuV, Arabidopsis, NLR, TIR‐NLR, TNL, *AT1G31540*, *GRP1*

## Abstract

Geminiviruses are single‐stranded DNA viruses that infect many plant species and cause serious losses in agronomically important crops. An earlier study showed that the *Arabidopsis thaliana* ecotype Pla‐1 is resistant to infection by diverse geminivirus species and mapped the major resistance locus *Geminivirus Resistance of Pla‐1 1* (*GRP1*) to chromosome 1. In this study, we fine‐mapped the *GRP1* locus to a 0.6‐Mb region and showed that its strength is gene‐dosage‐dependent. We also uncovered two minor resistance loci, *GRP2* and *GRP3*, that mapped to chromosomes 3 and 5, respectively, and showed that *GRP3* resistance is dependent on *GRP1*. RNA‐Seq analysis of plants inoculated with the geminivirus, cabbage leaf curl virus (CaLCuV, *Begomovirus brassicae*), showed that *AT1G31540,* which is located in the *GRP1* region and encodes a Toll/interleukin‐1 receptor (TIR) type nucleotide‐binding leucine‐rich repeat receptor (NLR), is upregulated in Pla‐1 compared to the susceptible Col‐0. *AT1G31540* specifies two TIR‐NLR isoforms that contain non‐synonymous codon differences between the two Arabidopsis ecotypes. Expression of the longer Pla‐1 isoform, which includes a dual‐segment leucine‐rich repeat domain and an integrated domain at the C terminus, conferred CaLCuV resistance to Col‐0, resulting in reduced viral DNA accumulation and no leaf chlorosis. In contrast, expression of the shorter isoform, which lacks the second leucine‐rich repeat segment and the integrated domain, did not confer resistance. This study established that effector‐triggered, TIR‐NLR‐mediated plant innate immunity contributes to geminivirus defense responses and identified a new host genetic resource to combat these important plant viral pathogens.

## Significance Statement

Geminiviruses are DNA viruses that cause serious diseases in plants and production losses in many important crops. This study identified a gene for a TIR‐NLR protein that confers resistance against geminivirus infection in Arabidopsis ecotype Pla‐1 and established that TIR‐NLR genes target geminiviruses during plant effector‐triggered immunity, expanding the resources available for combating agriculturally important geminivirus diseases.

## INTRODUCTION

Plants encounter biological stresses throughout their lives from fungal, bacterial, and viral pathogens, among others. Geminiviruses are a family of DNA viruses that infect both dicotyledonous and monocotyledonous plants (Jeske, 2009; Zhao et al., 2019). They often cause leaf yellowing or chlorosis, leaf curling and deformation, and stunted growth (Rojas et al., 2018). Geminivirus diseases can result in major yield reductions and, in some cases, total losses in important crops like cassava, tomato, cotton, and maize. Many crop plants and/or their wild relatives have evolved innate defense mechanisms that have been used in breeding programs to produce resistant varieties (Loriato et al., 2020; Shahriari et al., 2023). Increasing the genetic resources that can confer resistance or immunity against geminivirus infection will be critical for the control of current and future endemics caused by these important viral pathogens (Beam & Ascencio‐Ibanez, 2020; Hanley‐Bowdoin et al., 2013).

Geminiviruses are characterized by their small, circular single‐stranded DNA (ssDNA) genomes and their twinned icosahedral capsids. Begomoviruses, which constitute the largest and best‐studied genus in the Geminiviridae, infect dicotyledonous plants and are transmitted by whiteflies (*Bemisia tabaci*). Begomovirus genomes can consist of one DNA segment of approximately 3 kb in length or two genome segments of similar size (Zhao et al., 2019). The genome segments of bipartite begomoviruses, which are designated as DNA‐A and DNA‐B, are both required for infection and are packaged separately into virions (Bennett & Agbandje‐McKenna, 2020; Bottcher et al., 2004). Some begomoviruses are associated with smaller circular ssDNA satellites that play important roles in infection (Zhou, 2013). Begomovirus genomes encode 6–8 proteins, most of which are multifunctional and interact with host proteins (Fondong, 2013). These proteins, including C1 (Rep), C2 (TrAP), C3 (Ren), C4, V1 (CP), V2, BV1 (MP), and BV2 (NSP), are involved in viral replication, transcription, encapsidation, countering host defenses, and movement. Recent studies have indicated that the coding capacities of begomoviruses are greater than those of these canonical proteins (Chiu et al., 2022; Devendran et al., 2022; Gong et al., 2021).

Plants use many defense mechanisms against viral infection, and viruses use counter measures to circumvent host defenses (Hanley‐Bowdoin et al., 2013; Kumar, 2019; Zhang et al., 2023). Gene silencing pathways for both transcriptional gene silencing (TGS) and posttranscriptional gene silencing (PTGS) are key components of the host defenses against geminiviruses (Ceniceros‐Ojeda et al., 2016; Rodriguez‐Negrete et al., 2009). Two allelic genes (*Ty‐1* and *Ty‐3*) introgressed into tomato from a wild Solanaceous relative confer begomovirus resistance. *Ty‐1/Ty‐3* encode an atypical RNA‐dependent RNA polymerase that is involved in cytosine methylation of the viral genome during TGS and siRNA production during PTGS (Butterbach et al., 2014; Verlaan et al., 2013). Orthologous genes of *Ty‐1/Ty‐3* in pepper and cucumber were also found mediating geminivirus resistance (Koeda et al., 2022; Koeda et al., 2024). Posttranslational protein modifications have also been implicated in the host defense response against geminiviruses. The host SnRK1 kinase phosphorylates C2/TrAP, Rep and beta‐C1 (encoded by a satellite) to delay symptom development, and is countered by C2/TrAP, which inhibits SnRK1 kinase activity (Hao et al., 2003; Shen et al., 2011, 2014, 2018; Shen & Hanley‐Bowdoin, 2021). A receptor‐like protein kinase, NIK, interacts with the viral NSP and phosphorylates the host ribosomal RPL10 protein to suppress host and viral protein production (Martins et al., 2020). Mutations in host proteins that are necessary for the begomovirus life cycle have also been shown to confer resistance. Viral DNA replication requires the host DNA polymerase delta, and mutations in the polymerase delta subunit 1 gene have been implicated in cassava *CMD2* and tomato *Ty‐6* resistance against begomovirus infection (Lim et al., 2022; Shen et al., 2025; Wu et al., 2021). The *Ty‐5* locus, which confers recessive, broad‐based resistance in tomato, has a mutation in the host gene encoding the messenger RNA surveillance factor Pelota, which is involved in efficient protein translation (Koeda et al., 2021; Lapidot et al., 2015; Ren et al., 2022).

Plants have evolved two important innate immune systems that recognize, respond to, and limit invading pathogens (Ngou et al., 2022). Pathogen‐associated molecular pattern (PAMP)‐triggered immunity (PTI) involves cell surface receptors, mostly receptor‐like kinases (RLKs) and receptor‐like proteins (RLPs), that interact with pathogens extracellularly. Effector‐triggered immunity (ETI) involves a family of intracellular nucleotide‐binding/leucine‐rich repeat proteins (NLRs) that recognize pathogen effectors either directly or indirectly to counteract their virulence effects and trigger downstream defense processes (Contreras et al., 2023; Cui et al., 2015; Ngou et al., 2022; Sett et al., 2022; Teixeira et al., 2021; Wang et al., 2023). Activation of PTI or ETI through specific signaling pathways can lead to increased calcium influx, burst of reactive oxygen species and activation of transcription factors to reprogram host transcriptomes to contribute to pathogen defense, often resulting in programmed cell death known as the hypersensitive response (HR) to limit the spread of the pathogen. PTI and ETI are both involved in plant defenses against geminivirus infection (Calil & Fontes, 2017; Ray & Casteel, 2022; Sett et al., 2022). Transmembrane RLKs, like NIK that interacts with the viral protein NSP, have been shown to mediate resistance in Arabidopsis (Carvalho et al., 2008), while others have been implicated genetically, like an RLK gene from common bean that segregates with resistance to bean leaf crumple virus (Ariza‐Suarez et al., 2023).

NLR proteins usually have distinct N‐terminal domains including coiled coil (CC) or Toll/interleukin‐1 receptor (TIR) domains that are involved in the activation of downstream defense mechanisms against pathogen infection (Chia & Carella, 2023; Locci et al., 2023; Wang et al., 2023). The N‐terminal domain is followed by a nucleotide‐binding site (NBS) domain that binds and metabolizes nucleotides to activate the NLR protein (Wang et al., 2023). The NBS domain is followed by a leucine‐rich repeat (LRR) domain that interacts with and perceives pathogen effectors (Maruta et al., 2022; Wang et al., 2023). Some NLR proteins have integrated domains (IDs) at the C terminus or other locations in the protein that are also involved in effector recognition (Marchal et al., 2022; Maruta et al., 2022). The *Ty‐2* locus from the tomato relative *Solanum habrochaites* encodes a CC‐type NLR that confers resistance to tomato yellow leaf curl virus (TYLCV, *Begomovirus coheni*) (Yamaguchi et al., 2018). Another tomato CC‐NLR gene allele, *SlSw5a*, is responsible for the resistance to tomato leaf curl New Delhi virus (ToLCNDV, *Begomovirus solanumdelhiense*) by a cultivar and causes HR in infected plants (Sharma et al., 2021). Candidates for the viral effectors of *Ty‐2* and *SlSw5a* resistance include Rep and C4, respectively, which are encoded by overlapping open reading frames in begomovirus genomes (Sharma et al., 2021; Shen et al., 2020). A common bean (*Phaseolus vulgaris* L.) cultivar TIR‐NLR gene, *PvVTT1*, is upregulated in response to bean dwarf mosaic virus (BDMV, *Begomovirus moralesi*) infection and, when transiently expressed by agroinfiltration, confers BDMV resistance in a susceptible common bean cultivar (Seo et al., 2007). We report here the identification of a TIR‐type NLR allele from the *Arabidopsis thaliana* ecotype Pla‐1 that is required for endogenous resistance to cabbage leaf curl virus (CaLCuV, *Begomovirus brassicae*) infection and confers resistance to the susceptible Col‐0 ecotype when stably expressed from a transgene. Identification of the Pla‐1 TIR‐NLR resistance gene provides a new host genetic resource to combat begomovirus diseases.

## RESULTS

### The major genetic determinant for Pla‐1 CaLCuV resistance is dosage‐dependent

**Figure 1:**
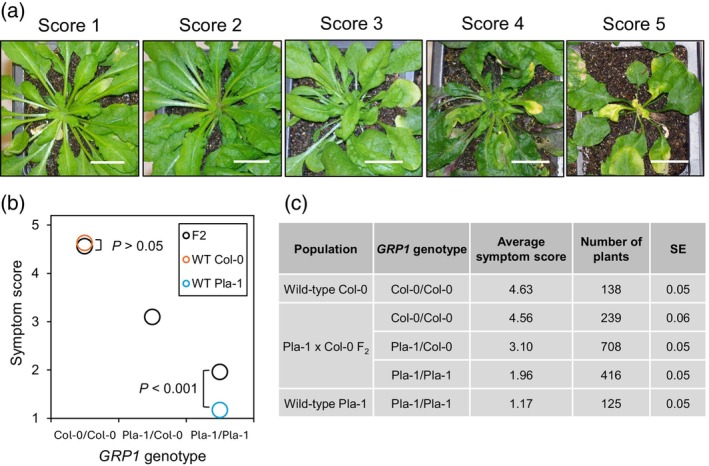
*GRP1* is required for resistance against CaLCuV infection in a dosage‐dependent manner. The genotypes of 2001 Pla‐1 × Col‐0 F_2_ plants at the two SNP positions of 8.0 and 12.7 Mb on chromosome 1 were determined by KASP. Plants with the same genotype at both SNPs were used as proxies for the *GRP1* genotypes. These plants, together with wild‐type (WT) Pla‐1 or Col‐0 plants, were grown and infected with CaLCuV in parallel. (a) Representative plants at 28 dpi with symptom scores from 1 to 5. Scale bar, 20 mm. (b, c) The symptom scores were recorded at 28 dpi, and the mean values for the three F_2_ genotypes and the wild‐type Pla‐1 and Col‐0 parents are shown. The population sizes and the standard errors (SE) for the mean scores are shown in panel (c). Student's *t*‐tests detected significant differences in the mean symptom scores between any of the F_2_ groups and between the *GRP1* ^*Pla‐1/Pla‐1*^ F_2_ plants and wild‐type Pla‐1 plants (*P* < 0.001). The mean scores of the *GRP1* ^*Col‐0/Col‐0*^ F_2_ plants and the wild‐type Col‐0 plants showed no significant difference (*P* > 0.05).

An earlier study showed that *Arabidopsis thaliana* ecotype Pla‐1 (referred to as Pla‐1 hereafter) displays near immunity to geminivirus infection and mapped the trait to a region between 8.0‐Mb and 12.7‐Mb on chromosome 1 (Reyes et al., 2017) (Genomic positions used throughout this paper correspond to the TAIR10 Col‐0 reference sequence from https://www.arabidopsis.org/). This locus was designated as *Geminivirus Resistance of Pla‐1 1* (*GRP1*). To further characterize and fine‐map *GRP1*, we generated a population of F_2_ plants from a cross between the resistant Pla‐1 and the susceptible Col‐0 ecotypes. We also used shotgun sequencing to re‐sequence the Pla‐1 genome to >200 × coverage to increase the number of single‐nucleotide polymorphisms (SNPs) between Pla‐1 and Col‐0 available for genetic mapping. F_2_ plants were infected with the begomovirus CaLCuV, and symptoms were recorded at 28 days post‐inoculation (dpi) using a scale from 1 to 5, in which 1 is no symptoms and 5 is severe symptoms (Figure 1a). PCR primers specific to two SNPs at the edges of the defined *GRP1* locus (chromosome 1 nucleotide positions 8 015 459 and 12 700 543) were used for the initial genotyping of F_2_ plants by Kompetitive allele specific PCR (KASP) (Table S1) (Semagn et al., 2014). This screen of 2001 F_2_ plants identified 416 plants homozygous for the Pla‐1 genotype at both SNP positions, 239 plants homozygous for the Col‐0 genotype at both SNP positions, and 708 plants heterozygous at both SNP positions (Figure 1c).

Using the three F_2_ populations as proxies for the genotypes of *GRP1*, we analyzed how *GRP1* contributes to the Pla‐1 resistance trait in CaLCuV infection assays. The susceptibility or resistance to geminivirus infection was treated as a quantitative trait using the symptom scores as nonparametric measurements. The mean symptom score of F_2_ plants homozygous for Pla‐1 *GRP1* was 1.96 at 28 dpi, which was significantly higher than the mean of 1.17 for wild‐type Pla‐1 plants (Figure 1b,c). The F_2_ plants homozygous for Col‐0 at *GRP1* had a mean symptom score of 4.56, which did not differ significantly from the mean for wild‐type Col‐0 plants, 4.63 (Figure 1b,c). These results indicated that Pla‐1 *GRP1* in the F_2_ plant population is necessary for resistance but not sufficient to confer immunity against geminivirus infection. The mean symptom score of the F_2_ plants heterozygous at the *GRP1* locus was 3.10, approximately in the middle of the two types of the homozygous plants, indicating that *GRP1* resistance is not dominant or recessive and, instead, depends on the dosage of the *GRP1* gene (Figure 1b,c).

### *GRP1* is fine‐mapped to a 0.6‐Mb region on Pla‐1 chromosome 1

**Figure 2:**
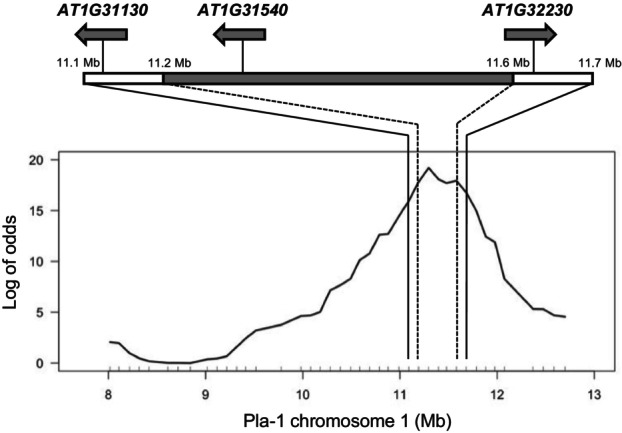
*GRP1* maps to a region between 11.1‐ and 11.7‐Mb on Pla‐1 chromosome 1. A population of 215 CaLCuV‐infected Pla‐1 × Col‐0 F_2_ plants heterozygous at one of the two SNP sites at 8.0 and 12.7 Mb of chromosome 1 and homozygous for Col‐0 at the other were used for QTL mapping. A set of intervening 39 SNPs, shown with inside tick marks, were selected for genotyping by KASP. CaLCuV symptoms were scored for each plant at 28 dpi. The Log of odds of QTL signals for resistance at each SNP were calculated using the nonparametric interval mapping method. The significance of the signal values set at *P* = 0.05 was estimated by a permutation test of 1000 replicas. The *GRP1* region was estimated by the 1.5‐unit decrease in Log of odds from the peak value (solid lines) or by the 95% Bayes credible interval (dashed lines). Shown on the top are the three gene loci detected by RNA‐Seq analysis that are more highly upregulated in CaLCuV‐inoculated leaves of Pla‐1 than Col‐0 plants.

We performed quantitative trait locus (QTL) analysis of the 8.0‐ to 12.7‐Mb region of chromosome 1 to fine map the Pla‐1 *GRP1* locus. Because *GRP1* is dosage‐dependent, we analyzed Pla‐1 × Col‐0 F_2_ plants containing a single partial copy of the Pla‐1 genome segment in this region. For these studies, we identified 215 plants homozygous for Col‐0 at one end and heterozygous for Pla‐1/Col‐0 at the other end in the KASP screen described above using the SNPs at positions 8 015 459 and 12 700 543. This population of plants, which resembled progenies of F_1_ hybrid plants backcrossed to the parental Col‐0, were analyzed using CaLCuV symptom scores as quantitative traits for genetic mapping. A set of 41 SNPs, including the two at the edge positions of the region, spaced at approximately 100‐kb intervals were used as markers for KASP genotyping (Table S1). The R‐based software R/qtl (Broman & Sen, 2009) was used to calculate the log of odds (LOD) of genotype–phenotype correlation at each SNP site (Figure 2). A strong, significant QTL signal spanning much of the region peaked at SNP position 11 296 101. The chromosome 1 segment containing the gene responsible for *GRP1* resistance was mapped with 95% confidence between the markers at positions 11 191 288 and 11 581 529 using Bayes credible interval analysis (Figure 2). The *GRP1* locus was mapped between positions 11 091 262 and 11 680 908 using a 1.5 decrease in LOD from the peak value. This assessment corresponds to a 0.6‐Mb region on the Pla‐1 chromosome 1 around the peak at position 11.3‐Mb (Figure 2).

### There may be other Pla‐1 loci involved in the resistance phenotype

A previous attempt to identify other *GRP* loci that might be involved in resistance was not successful due to the low coverage of the available SNP markers between the Pla‐1 and Col‐0 genomes (Reyes et al., 2017). In the present study, we used the sensitive QTL‐Seq method to re‐map geminivirus resistance in the Pla‐1 genome (Takagi et al., 2013). From a population of Pla‐1 × Col‐0 F_2_ plants infected with CaLCuV, two subpopulations of 50 plants each were assembled. One subpopulation included asymptomatic plants (symptom score = 1), while the other included plants showing severe symptoms (symptom score = 5). Genomic DNA was isolated from each F_2_ plant separately. Equal amounts of each of the 50 DNA samples were pooled for each subpopulation and used for Illumina sequencing. The sequencing reads from the two populations were aligned to the Col‐0 genome sequence. The average genomic sequencing depth was 140× for the symptomless F_2_ plants and 133× for the plants with the most severe scores.

**Figure 3:**
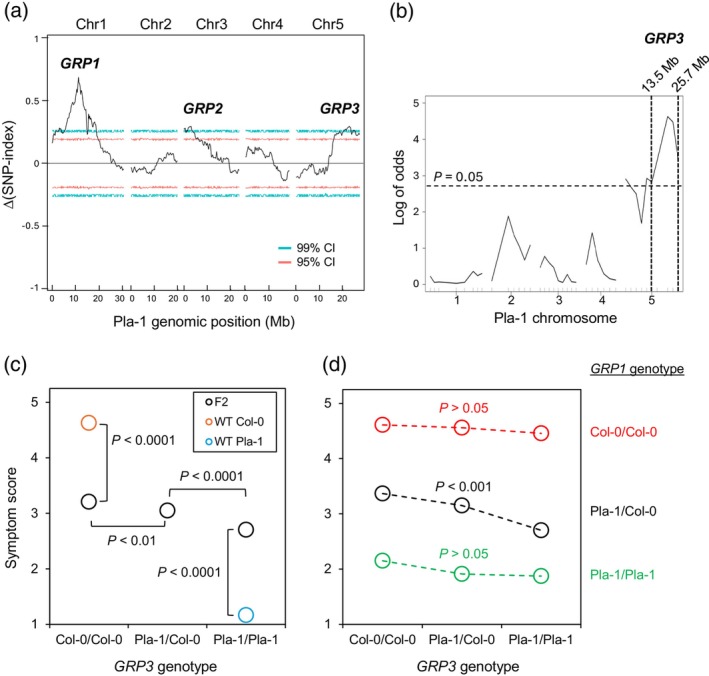
The Pla‐1 genome contains minor *GRP* loci that contribute to geminivirus immunity. (a) QTL‐Seq analysis of bulks DNA samples from 50 Pla‐1 × Col‐0 F_2_ plants with no symptoms (score 1) and 50 Pla‐1 × Col‐0 F_2_ plants showing severe symptoms (score 5) after CaLCuV inoculation. Differences between the SNP indexes of the Pla‐1 sequence content derived from 3 846 180 SNPs confirmed the major *GRP1* resistance locus in the Pla‐1 chromosome 1 and revealed two minor loci, *GRP2* in chromosome 3 and *GRP3* in chromosome 5, beyond confidence intervals (CIs) of 99%. (b) *GRP* *3* was confirmed by KASP genotyping and QTL mapping using 344 CaLCuV‐infected Pla‐1 × Col‐0 F_2_ plants that were heterozygous at the *GRP1* locus and mapped to a region on chromosome 5 from 13.5 to 25.7 Mb using the criterion of 1.5‐unit decrease in Log of odds from the peak value. The analysis used selected SNP markers (inside tick marks) at 2.5‐Mb intervals across the Arabidopsis genome. (c) The contribution of *GRP3* to Pla‐1 resistance to CaLCuV infection was estimated using 2154 Pla‐1 × Col‐0 F_2_ plants of various genotypes at the SNP at 20.8 Mb of chromosome 5 as a proxy for the *GRP3* genotype. The mean symptom scores at 28 dpi for the three *GRP3* genotypes and the wild‐type (WT) Pla‐1 and Col‐0 parents are shown. The population sizes and the standard errors for the mean scores are shown in Figure S1(a). Student's *t*‐tests detected significant differences in mean symptom scores in the indicated comparisons. (d) The mean symptom scores of each of the combinations of *GRP1* and *GRP3* genotypes of the CaLCuV‐infected Pla‐1 × Col‐0 F_2_ plants are shown for interaction of *GRP1* and *GRP3* in contributions to geminivirus resistance. The population sizes and the standard errors for the mean scores are shown in Figure S1(b). The *P* values are from ANOVA tests performed between the *GRP3* genotypes within each *GRP1* genotype group.

We called 3 846 180 SNPs between the Pla‐1 and Col‐0 genomes using 137 818 086 aligned paired‐end 150‐bp reads. SNP indexes of the Pla‐1 sequence content in the bulk DNA from symptomless plants and the Col‐0 sequence content in the bulk DNA from severely infected plants were calculated at each SNP (Takagi et al., 2013). The difference of the two indexes is a measurement of QTL signals for Pla‐1 resistance. Figure 3(a) shows a plot of the average differences in the indexes using a 300‐kb sliding window with a 10‐kb fixed step length along the Col‐0 genome. Three peaks with >99% confidence intervals were observed. A major peak occurred at the SNP at position 11 407 413 on chromosome 1, corresponding well to the *GRP1* locus mapped above (Figure 3a). Two less prominent peaks centered on SNPs at position 2 200 578 on chromosome 3 and position 22 336 271 on chromosome 5. We designated these peaks as *GRP2* and *GRP3*, respectively (Figure 3a).

The improved Pla‐1 genome sequence enabled us to verify the two minor QTLs by KASP analysis using selected SNP markers between Pla‐1 and Col‐0 covering the entire genome at 2.5‐Mb intervals (Table S2). Because *GRP1* is required for the resistance, we analyzed Pla‐1 × Col‐0 F_2_ plants heterozygous for *GRP1* to increase mapping sensitivity. A population of 344 F_2_ plants of the genotype *Pla‐1/Col‐0* at both 8.0 and 12.7 Mb was used as a proxy for the heterozygous *GRP1* ^*Pla‐1/Col‐0*^, assuming the recombination rate in this short region is very low. Mapping with this population gave a weak QTL signal near the end of chromosome 5 with a peak corresponding to the SNP at position 20 800 880 (Figure 3b). The span of *GRP3* mapped between positions 13 462 453 and 25 708 060 using the criterion of decrease in LOD by 1.5 from the peak value. The 95% Bayes credible interval was slightly narrower, ranging from 15 907 450 to 25 708 060 (Figure 3b). In contrast, the marker‐based mapping did not give any significant signals of the *GRP2* locus (Figure 3b). Together, these analyses confirmed that *GRP3*, but not *GRP2*, contributes to the resistance phenotype.

To investigate how the *GRP3* locus contributes to Pla‐1 resistance, we analyzed the relationship between the *GRP3* genotypes of the Pla‐1 × Col‐0 F_2_ plants and resistance to CaLCuV infection regardless of the genotype of *GRP1*. We used the genotypes of the SNP at position 20 800 880 on chromosome 5 as proxies of the *GRP3* genotypes (Table S2). In the population of 2154 F_2_ plants with determined genotypes at this position, 475 *GRP3* ^*Pla‐1/Pla‐1*^, 1118 *GRP3* ^*Pla‐1/Col‐0*^, and 561 *GRP3* ^*Col‐0/Col‐0*^ plants showed average symptom scores of 2.7, 3.1 and 3.2, respectively (Figure 3c; Figure S1a). The scores for heterozygous *GRP3* plants were significantly higher than those of plants homozygous for Pla‐1 at *GRP3* and significantly lower than those of plants homozygous for Col‐0 at *GRP3*. These results showed that the presence of Pla‐1 *GRP3* enhances geminivirus resistance in a dosage‐dependent manner. However, two observations indicated that the contribution of *GRP3* to resistance is small and not essential (Figure 3c; Figure S1a). First, the average symptom scores of *GRP3* ^*Pla‐1/Pla‐1*^ plants were much higher than those of wild‐type Pla‐1 plants. Second, *GRP3* ^*Col‐0/Col‐0*^ plants with no Pla‐1 *GRP3* sequences showed substantial resistance against CaLCuV.

We also asked if there are interactions between the different *GRP3* and *GRP1* genotypes in F_2_ plants (Figure 3d; Figure S1b). The presence of one or two copies of the Pla‐1 *GRP3* allele only enhanced resistance to CaLCuV when *GRP1* was heterozygous. Pla‐1 *GRP3* did not alter resistance when the *GRP1* locus was homozygous for either Pla‐1 or Col‐0. The different effects of *GRP3* on plants with one or two copies of the Pla‐1 *GRP1* allele likely reflect the dose‐responsive nature of *GRP1* resistance. The very low symptom scores of homozygous *GRP1* ^*Pla‐1*^ plants (similar to wild‐type Pla‐1) would make it difficult to detect a small effect of *GRP3* on resistance. These results are consistent with *GRP1* being epistatic to *GRP3*. Moreover, the dosage‐dependent effects of both loci on geminivirus resistance suggest that the expression levels of both genes determine the resistance outcome.

### Identification of genes in the *GRP1* locus that are differentially regulated by CaLCuV infection in Pla‐1 and Col‐0

The 0.6‐Mb region on chromosome 1 that encompasses *GRP1* contains 134 protein coding genes according to the TAIR10 Col‐0 reference genome sequence (Table S3; Figure S2). To identify which gene(s) in the *GRP1* locus confer resistance, we assumed that *GRP1* resistance is due to a gain‐of‐function mutation that alters the amino acid sequence of a protein. We also hypothesized that the *GRP1* resistance gene is more highly expressed in Pla‐1 than its allele in Col‐0 and may be upregulated by geminivirus infection. To test our hypotheses, we used RNA‐Seq to compare Pla‐1 and Col‐0 gene expression profiles in untreated leaves or in leaves inoculated with CaLCuV or an empty vector to control for the effects of biolistic inoculation. Inoculated leaves were sampled at 4 dpi because an earlier study showed that viral DNA does not accumulate in inoculated leaves of Pla‐1 plants, indicating that resistance occurs early in the infection process (Reyes et al., 2017). The experimental design included four biological replicates for each of the three treatments in the two genotypes. No expression was detected for 48 of the protein coding genes for either Pla‐1 or Col‐0 in any of the three treatments and they were excluded as candidates for *GRP1* (Figure S2). Expression was detected for the remaining 86 genes in at least one genotype and treatment and 56 of them specify protein products with amino acid polymorphisms between Pla‐1 and Col‐0 (Table S4; Figure S2). One gene locus is disrupted in Pla‐1 and produced no transcripts in Pla‐1 (Table S4; Figure S2). To recover potential missing regions in Col‐0, we also aligned the RNA‐Seq reads with the genome sequence of the ecotype Ler‐0, which is not genetically closely related to Col‐0 among the Arabidopsis ecotypes (Lee et al., 2014), but did not uncover any additional expressed Pla‐1 genes (data not shown).

Many of the expressed genes in the *GRP1* locus showed differential gene expression (DGE) between Pla‐1 and Col‐0 and in response to CaLCuV using an adjusted *P* < 0.05 cutoff. Comparison of healthy plants detected higher transcript levels for 24 genes in Pla‐1 and for 22 genes in Col‐0 (Table S4). DGE analysis of the responses to CaLCuV was first analyzed separately in Pla‐1 and Col‐1 by comparing expression profiles from the CaLCuV inoculation and mock treatments in each genotype. In Pla‐1, 12 and 23 genes were upregulated and downregulated, respectively, by CaLCuV inoculation. In Col‐0, CaLCuV inoculation resulted in the upregulation and downregulation of two and five genes, respectively (Table S4). After using the mock control to adjust for inoculation effects, comparison of Pla‐1 versus Col‐0 identified six genes in the *GRP1* locus that were differentially regulated in response to CaLCuV in the two genotypes. Three were more highly expressed in Pla‐1 and 3 were more highly expressed in Col‐0 (Table S4).

**Figure 4:**
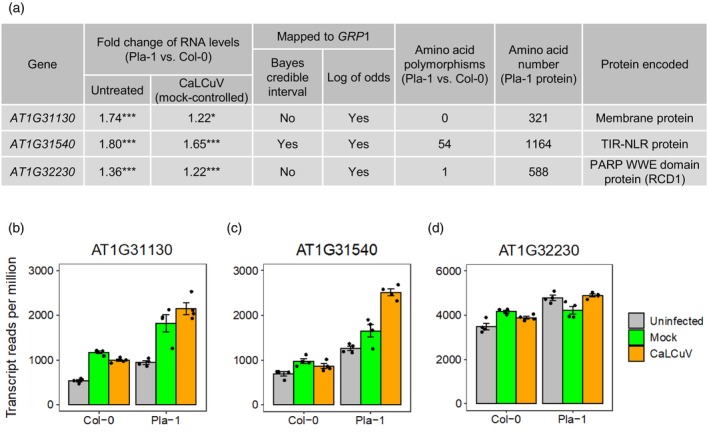
RNA‐Seq analysis of genes in the *GRP1* locus. (a) RNA‐Seq was performed on Pla‐1 or Col‐0 plants that were untreated, inoculated with CaLCuV, or mock inoculated (for control) with four bioreplicates per treatment. The RNA was isolated from inoculated leaves at 4 dpi and from equivalent leaves for untreated plants. The Illumina sequencing reads were aligned to the Col‐0 genome and analyzed for differential gene expression. The genes within the mapped *GRP1* locus showing higher expression levels in Pla‐1 than in Col‐0 when infected with CaLCuV are listed. The fold‐change values shown are all significant (*Adjusted *P* < 0.05; ***Adjusted *P* < 0.001). The number of amino acid polymorphisms in the proteins encoded by the Pla‐1 and Col‐0 alleles and the total number of amino acid residues in the Pla‐1 proteins are shown. (b–d) The means and standard errors of the normalized read counts (reads per million) for the three genes are shown for the different treatments (*n* = 4).

The three genes that were upregulated in Pla‐1 in response to CaLCuV are *AT1G31130*, *AT1G31540*, and *AT1G32230* (Figure 4). *AT1G31540* and *AT1G32230*, but not *AT1G31130*, also showed increased transcript levels in the comparison of virus‐inoculated versus mock‐inoculated Pla‐1 plants. Healthy Pla‐1 plants expressed the three genes at higher levels than healthy Col‐0 plants. All three genes are included in the *GRP1* locus defined by the LOD test. However, only *AT1G31540* occurs in the *GRP1* locus when the more stringent 95% Bayes credible interval was used to define the locus to a narrower region between positions 11 191 288 and 11 581 529 in Pla‐1 chromosome 1 (Figure 2; Figure 4a). We eliminated *AT1G31130* from further consideration because the protein products encoded by the Pla‐1 and Col‐0 alleles are identical. The proteins encoded by *AT1G31540* or *AT1G32230* have amino acid polymorphisms (Figure 4a; Figure S3) that could contribute to the resistant and susceptible responses to CaLCuV in Pla‐1 and Col‐0, respectively. *AT1G31540* encodes a TIR‐NLR protein (Figure S3), and *AT1G32230* encodes the Radical‐Induced Cell Death 1 (RCD1) protein that belongs to the (ADP‐ribosyl) transferase domain‐containing subfamily of the WWE protein–protein interaction domain family. Both of these classes of proteins have been implicated in the plant defense response in other pathosystems (Cui et al., 2015; Ngou et al., 2022; Sett et al., 2022; Wirthmueller et al., 2018), making them good candidates for the *GRP1* gene in Pla‐1.

### The Pla‐1 *AT1G31540.2* NLR gene confers resistance to CaLCuV infection in Col‐0

According to the TAIR10 annotations (https://www.arabidopsis.org/), the *AT1G31540* locus specifies three gene models resulting in two protein products. The *AT1G31540.2* gene model encodes a long version of the TIR‐NLR protein, while the proteins encoded by *AT1G31540.1* and *AT1G31540.3* specify identical, shorter versions with C‐terminal truncations (Figure S3). The *AT1G32230* gene has six gene models with five (*AT1G32230.1*, *AT1G32230.2*, *AT1G32230.4*, *AT1G32230.5* and *AT1G32230.6*) encoding identical proteins except for an extra Gln residue at the 5′ end of the fourth exon in AT1G32230.1. The protein specified by *AT1G32230.3* is slightly shorter at the C‐terminus. The Pla‐1 genomic sequences of these two genes are similar to the Col‐0 sequences, especially around the translational start and stop codons and the exon‐intron junction sites, and the gene models and protein products deduced from Col‐0 are also likely to apply to Pla‐1.

**Figure 5:**
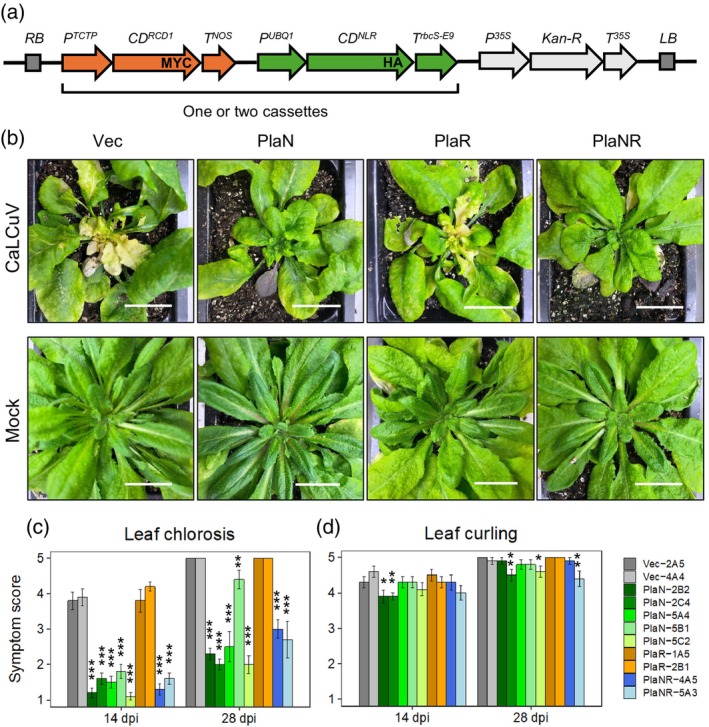
Expression of the Pla‐1 *AT1G31540* NLR gene confers CaLCuV resistance to susceptible Col‐0 plants. (a) Diagram of the Pla‐1 gene expression cassettes. The Pla‐1 *AT1G31540.2* (*NLR*) coding sequence has an HA tag at the 3′ end and is flanked by *UBQ1* promoter and the *rbcs‐E9* terminator sequences. The Pla‐1 *AT1G32230.1* (*RCD1*) coding sequence has an MYC tag at the 3′ end and is flanked by *TCTP* promoter and the *NOS* terminator sequences. The expression cassettes were cloned individually or together upstream of kanamycin selectable marker (Kan‐R) and transformed into Col‐0. PlaN lines contain the AT1G31540.2 expression cassette, PlaR lines contain the AT1G32230.1 expression cassette, and PlaNR lines contain both. Vec lines contain the vector without any expression cassettes. (b) Images of agroinoculated CaLCuV‐ or mock‐inoculated T_3_ transgenic plants carrying an individual or dual cassette at 28 dpi. One representative line of each type of the transformants is displayed. The lines shown are Vec‐4A4, PlaN‐5C2, PlaR‐1A5 and PlaNR‐5A3. Scale bar, 20 mm. (c, d) Average symptom scores for leaf chlorosis (c) and leaf curling (d) at 14 and 28 dpi for two Vec lines, five PlaN lines, two PlaR lines, and two PlaNR lines (*n* = 10). Transgenic lines showing significant differences in symptom scores from the Vec lines as assessed by the Mann–Whitney test are indicated with asterisks (*P < 0.05; **P < 0.01; ***P < 0.001). The bars indicate standard errors.

We used the Col‐0 gene models to design expression cassettes for the homologous Pla‐1 genes. For locus *AT1G31540*, we cloned the cDNA for the gene model *AT1G31540.2* that codes for the long TIR‐NLR isoform. For locus *AT1G32230*, we cloned a cDNA for the gene model *AT1G32230.1* with an extended C‐terminus. The successful cDNA cloning using Pla‐1 RNA confirmed the general applicability of the Col‐0 gene models. The Arabidopsis *UBQ1* promoter (Holtorf et al., 1995) and the *rbcS‐E9* terminator were used for expression of the Pla‐1 AT1G31540.2 cassette (designated as PlaN lines). The Arabidopsis *TCTP* promoter (Han et al., 2015) and the *NOS* terminator were used for the Pla‐1 AT1G32230.1 cassette (PlaR lines) (Figure 5a). We also made a construct containing both gene expression cassettes in tandem (PlaNR lines) (Figure 5a) to address the possibility that both genes are required for resistance. Col‐0 plants were transformed with the three Pla‐1 expression cassettes, and T_3_ homozygous lines with single transgene insertion sites were used to test for CaLCuV resistance. We also generated control lines transformed with an empty T‐DNA vector (Vec lines). Transgene expression was confirmed in selected representative lines containing the different cassettes (Figure S4).

The transgenic Col‐0 lines were agroinoculated with CaLCuV, and plants were monitored for leaf chlorosis and leaf curling – symptoms that are characteristic of infection. Initial studies indicated the PlaN and PlaNR lines, which contain Pla‐1 *AT1G31540.2* expression cassettes, developed very mild leaf chlorosis, while the PlaR and Vec lines displayed extensive chlorosis and bleaching (Figure 5b). In contrast, leaf curling was similar for all the transgenic lines, including the Vec lines (Figure 5b). Given these results, we scored leaf chlorosis and leaf curling separately in subsequent experiments using scales from 1 (no symptoms) to 5 (severe symptoms). In Figure 5(c), the average chlorosis scores for CaLCuV‐inoculated PlaN plants were low at 14 dpi (1.1–1.6) and 28 dpi (2.0–2.3). Even though PlaN‐5B1 plants had an average chlorosis score of 4.3 at 28 dpi, this value was still significantly lower (*P* value <0.05) than the Vec control lines with scores of 5 at 28 dpi. The two PlaNR lines also had lower average chlorosis scores at 14 and 28 dpi, while the PlaR lines had scores indistinguishable from the Vec control lines. The differences in the average leaf curling scores were much smaller across the various transgenic lines (Figure 5d). Some PlaN and PlaNR lines had leaf curling scores that were statistically lower than the Vec controls, but the differences were small relative to the large effects seen for leaf chlorosis (Figure 5c,d). These results established that expression of the Pla‐1 *AT1G31540.2*, but not Pla‐1 *AT1G32230.1*, in susceptible Col‐0 plants greatly reduced leaf chlorosis and had a small effect on leaf curling. This analysis excluded the Pla‐1 *AT1G32230* gene as a candidate for *GRP1*.

In the above experiments, CaLCuV was inoculated using Agrobacteria containing Ti plasmids with the viral DNA replicons inserted into the T‐DNA. NLR proteins have been widely reported to confer plant resistance to bacterial pathogens (Cui et al., 2015; Ngou et al., 2022), so it was important to rule out that Pla‐1 *AT1G31540.2* confers CaLCuV resistance by interfering with the ability of Agrobacteria to transfer viral DNA replicons into Arabidopsis. To address this possibility, we used biolistic inoculation, which does not depend on T‐DNA transfer to deliver viral replicons into the transgenic Col‐0 lines, and monitored symptoms overtime. PlaN lines showed no leaf chlorosis before 15 dpi and only mild chlorosis at 28 dpi (Figure S5). In contrast, wild‐type Col‐0 and the Vec control lines displayed severe chlorosis at 12 dpi. As observed in earlier experiments, reduction in leaf curling was minimal in the PlaN lines compared to the control lines (Figure S5). Together, these results established that Pla‐1 *AT1G31540.2* confers resistance to CaLCuV infection and does not interfere with *Agrobacterium*‐mediated inoculation of the virus.

### The long isoform of Pla‐1 AT1G31540 is required for CaLCuV resistance

**Figure 6:**
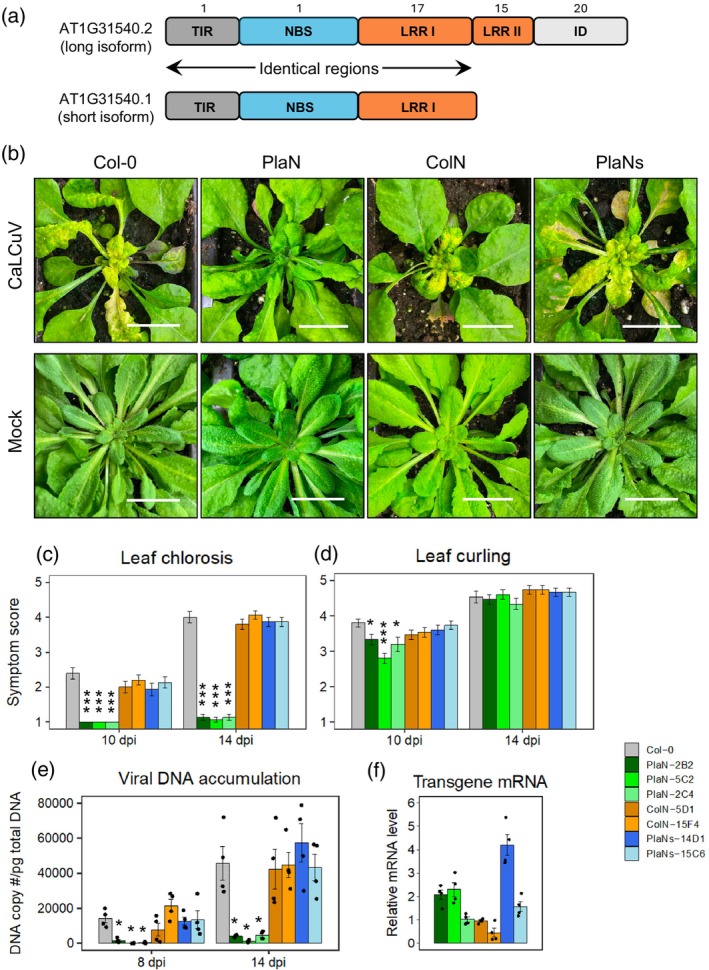
The long isoform of the Pla‐1 *AT1G31540* NLR gene suppresses CaLCuV DNA accumulation and symptom development. (a) Diagram of the long and short isoforms of the Pla‐1 and Col‐0 AT1G31540 NLR homologs. The protein domains are Toll‐like/interleukin (TIR), nucleotide‐binding site (NBS), leucine‐rich repeats (LRR) I and II, and integrated domain (ID). The numbers of amino acid differences in each domain of the long isoform between Pla‐1 and Col‐0 are indicated at the top. (b) Images of agroinoculated CaLCuV‐ or mock‐inoculated T_3_ plants carrying transgenes for the long Pla‐1 isoform (PlaN; AT1G31540.2), the long Col‐0 isoform (ColN; AT1G31540.2), or the short Pla‐1 isoform (PlaNs; AT1G31540.1) at 21 dpi. One representative line of each type of the transformants and wild‐type Col‐0 is displayed. The lines are PlaN‐5C2, ColR‐5D1 and PlaNs‐15C6. Scale bar, 20 mm. (c, d) Average symptom scores for leaf chlorosis (c) and leaf curling (d) at 10 and 14 dpi for wild‐type Col‐0, three PlaN lines, two ColN lines, and two PlaNs lines (*n* = 10). Transgenic lines showing significant differences in symptom scores from wild‐type Col‐0 plants as assessed by the Mann–Whitney test are indicated with asterisks (*P < 0.05; ***P < 0.001). (e) The copy number of CaLCuV DNA‐A in total DNA samples from young rosette leaves and the shoot apical meristems of infected plants was determined at 8 or 14 dpi using qPCR and a standard curve generated from a plasmid containing the DNA‐A sequence. The mean DNA‐A copies per picogram of total input DNA are shown (*n* = 4). The asterisks indicate significant difference from the wild‐type Col‐0 plants as determined by Student's *t*‐tests (*P* < 0.05). The bars indicate standard errors. (f) Transgene expression levels were assessed by RT‐qPCR of RNA from untreated plants. The cDNA copy numbers were measured using standard curves derived from the cloned transgenes and normalized against the expression levels of the *AP2M* reference gene. The RNA level of the line PlaN‐2C4 was arbitrarily set at 1 for comparison with the other lines when the relative RNA levels are converted to linear scales. The mean transcript levels and their standard errors were shown (*n* = 4).

The Pla‐1 and Col‐0 *AT1G31540* genes map to the Col‐0 chromosome 1 nucleotide positions 11 289 247 to 11 293 696 in the antisense orientation (Figure 2). TAIR10 includes three gene models for AT1G31540 that together specify two isoforms of a TIR‐type NLR protein. The AT1G31540.2 model describes a long isoform that encodes 1164 and 1161 amino acid residues in Pla‐1 and Col‐0, respectively (Figure S3a). The long isoforms have similar domain structures, with TIR domains at their N‐termini followed by NBS domains, two LRR domain segments (LRR I and LRR II), and IDs at their C‐termini (Figure 6a; Figure S3a). Many NLRs contain IDs after their LRR domains (Marchal et al., 2022), and the two domains often mediate effector recognition (Maruta et al., 2022). The AT1G31540.1 and AT1G31540.3 models describe a short isoform that lacks the LRR II segment and ID (Figure S3).

The Pla‐1 and Col‐0 long isoforms display 95.4% amino acid identity. The TIR and NBS domains of both isoforms each contain a single polymorphism, while the LRR and ID domains of the long isoform contain 32 and 15 amino acid differences, respectively (Figure 6a; Figure S3a). The Pla‐1 ID also has two 2‐residue insertions and one 1‐residue deletion compared to the Col‐0 ID (Figure S3a). The truncated LRR domain of the short isoform has 19 amino acid differences (Figure S3). Several SNPs and short insertions occur in the 1‐kb sequence upstream of the translation start site of the Pla‐1 *AT1G31540* gene compared to the Col‐0 allele (not shown).

The existence of two GRP1 isoforms and the high level of amino acid identity between the Pla‐1 and Col‐0 homologs raised two questions about the requirements for resistance. First, can the Pla‐1 AT1G31540.1 short isoform lacking the LRR II segment and ID confer virus resistance to Col‐0? Second, can the Col‐0 AT1G31540.2 long isoform, which has the same domain structure and a related amino acid sequence to the Pla‐1 long isoform, confer resistance when expressed at higher levels under the control of the *UBQ1* promoter?

We cloned cDNAs for the Pla‐1 short isoform (AT1G31540.1) and the Col‐0 long isoform (AT1G31540.2), confirming their endogenous expression. Both coding regions with HA‐tags at their 3′ ends were cloned into the expression cassette with the *UBQ1* promoter and the *rbcS‐E9* terminator – the same cassette used for the Pla‐1 long isoform. Col‐0 plants were transformed with the new cassettes, and T_3_ homozygous lines with single T‐DNA insertions were obtained. We selected three lines with the Pla‐1 long isoform (PlaN), two lines with the Pla‐1 short isoform (PlaNs), and two lines with the Col‐0 long isoform (ColN) for analysis of transgene expression by RT‐qPCR. The transgenes were expressed in all the selected lines (Figure 6f). The PlaNs‐14D1 transgene was expressed twofold higher than the highest expressing PlaN transgene, and PlaNs‐15C6 transgene expression was higher than PlaN‐2C4. ColN‐5D1 and PlaN‐2C4 had similar levels of expression.

When the T_3_ lines were challenged with CaLCuV, all the lines expressing the Pla‐1 short isoform (PlaNs lines) or the Col‐0 long isoform (ColN lines) of the AT1G31540 NLR protein displayed leaf chlorosis and leaf curling symptoms that were indistinguishable from wild‐type Col‐0 plants (Figure 6b−d). In contrast, lines expressing the Pla‐1 long isoform (PlaN lines) remained green and had very low chlorosis symptom scores (Figure 6b−d). Some PlaN plants showed slightly less leaf curling at 10 dpi relative to wild‐type Col‐0 plants, but there was no difference in the leaf curling symptoms at 14 dpi (Figure 6d).

We also measured viral DNA accumulation in the infected plants using qPCR to determine the copy number of CaLCuV DNA‐A. Viral DNA was detected in wild‐type Col‐0 plants and in plants representing all the transgenic lines at 8 and 14 dpi (Figure 6e). Viral DNA accumulation was similar in the PlaNs lines, ColN lines and wild‐type Col‐0 plants. In contrast, the PlaN lines accumulated from 9 to 115‐fold and 10 to 34‐fold less viral DNA than wild‐type Col‐0 plants at 8 and 14 dpi, respectively. These results established that the long isoform of the Pla‐1 AT1G31540 TIR‐NLR protein is required to confer resistance to CaLCuV infection in Col‐0. Moreover, the strong reduction in viral DNA accumulation by the Pla‐1 long isoform in the PlaN lines correlated with the suppression of leaf chlorosis but was not sufficient for suppression of leaf curling.

We also examined transgene protein production in uninfected plants by protein immunoprecipitation with immobilized HA antibodies followed by immunoblotting on protein gels. Both the long and short isoforms of the Pla‐1 AT1G31540 proteins, produced in the PlaN and PlaNs lines, respectively, were detected on immunoblots. However, we were unable to detect the long isoform of the Col‐0 AT1G31540 protein in the ColN lines analyzed in parallel with the Pla‐1 orthologs (Figure S6). Given that transcripts for all three forms of the transgenes were readily detectable (Figure 6f), the absence of the Col‐0 AT1G31540 transgene protein suggests that it is unstable or translated inefficiently.

## DISCUSSION

Diseases caused by geminiviruses have emerged as serious threats to many crop species (Fiallo‐Olive & Navas‐Castillo, 2023; Navas‐Castillo et al., 2011), but only a limited number of geminivirus resistance genes have been identified and used in breeding programs. Examples include the tomato *Ty* genes introgressed from wild relatives and the cassava *CMD2* genes found in some cultivars (Beam & Ascencio‐Ibanez, 2020; Fondong, 2017; Gill et al., 2019; Lim et al., 2022). Arabidopsis could be an important source of host resistance against plant pathogens because of its wide ecological distribution and high genetic variability (Kang et al., 2023). This potential is illustrated by the Pla‐1 ecotype, which was highly resistant to geminivirus infection in a screen of more than 200 Arabidopsis accessions (Reyes et al., 2017). In this report, we identified a TIR‐NLR gene as the major resistance determinant against geminivirus infection in Pla‐1 and showed that a Pla‐1 TIR‐NLR transgene confers the resistance phenotype to a susceptible Arabidopsis ecotype. The Pla‐1 TIR‐NLR gene is a new genetic resource that can be included in the small arsenal of host genes that combat geminivirus diseases.

Our previous study identified a major QTL for resistance to CaLCuV infection on Pla‐1 chromosome 1 (Reyes et al., 2017). In this report, the QTL was mapped to a 0.6‐Mb region designated as the *GRP1* locus (Figure 2), which contains 155 annotated genes that are candidates for the *GRP1* gene(s). Genes involved in responding to and countering biotic or abiotic stresses are often upregulated in response to the stress (Yang et al., 2021). Moreover, it was more likely that a differentially expressed gene with non‐synonymous codon changes would confer resistance than a gene encoding a conserved protein. Only two genes in the *GRP1* locus, *AT1G31540* and *AT1G32230*, met both these criteria (Figure 4). Transgenic experiments established that the Pla‐1 allele of *AT1G31540* confers CaLCuV resistance in the susceptible ecotype, Col‐0 (Figure 5), and that *AT1G32230* has no detectable role in resistance.

Genetic analysis of Pla‐1 × Col‐0 F_2_ plants established that *GRP1* is necessary for geminivirus resistance and that resistance is proportional to its copy number in the F_2_ population (Figure 1b,c). However, F_2_ plants homozygous for the Pla‐1 *GRP1* allele were less resistant than wild‐type Pla‐1 plants. We found two minor genetic loci on other chromosomes that contribute to Pla‐1 resistance and, as such, can segregate away from the *GRP1* locus in F_2_ plants (Figure 3a,b). These loci could contribute independently or in combination with *GRP1* to produce wild‐type levels of resistance. There is precedence in plants for modifier genes that augment resistance against viral pathosystems (Gallois et al., 2018; Larsen & Miklas, 2004). We examined the relationship between *GRP1* and *GRP3*, which had the strongest secondary QTL signal in Pla‐1 × Col‐0 F_2_ plants. This analysis showed that *GRP3* requires the presence of *GRP1*, while *GRP1* can sustain resistance in the absence of *GRP3* (Figure 3d). Hence, *GRP3* is likely a genetic modifier of *GRP1*, possibly encoding a transcription factor that enhances the expression level of *GRP1* in Pla‐1 or a protein that maintains the stability of the GRP1 protein in the cell. The gene dosage dependency of *GRP1* resistance underscores the importance of expression level in conferring strong resistance. The *GRP3* QTL maps to a very wide region on Pla‐1 chromosome 5 that includes many genes (Figure 3a,b). Because of the dependency of *GRP3* activity on *GRP1* resistance, a much larger Pla‐1 × Col‐0 F_2_ population than was used for *GRP1* mapping would be required to identify the *GRP3* gene and assess fully its role in resistance.

The response of Col‐0 plants expressing the *GRP1* transgene to CaLCuV infection is complex. In wild‐type Col‐0, CaLCuV infection is associated with severe symptoms including leaf curling and chlorosis – two major symptoms often associated with geminivirus infections (Navas‐Castillo et al., 2011). Both T_2_ (not shown) and T_3_ plants (Figures 5b,c and 6b,c) expressing *GRP1* showed a reduction in CaLCuV symptoms, establishing that the Pla‐1 allele of *AT1G31540* is sufficient to confer resistance in a susceptible background. However, the transgenic *GRP1* plants did not achieve the same level of resistance as wild‐type Pla‐1 plants (Reyes et al., 2017), consistent with the presence of other loci in the Pla‐1 genome contributing to resistance. Homozygous T_3_ *GRP1* plants showed a small reduction in leaf curling early in the infection process but did not differ significantly from infected wild‐type plants at later times (Figures 5b,d and 6b,d). In contrast, the development of chlorosis was strongly suppressed throughout infection in *GRP1* plants (Figures 5b,c and 6b,c). This suppression paralleled viral accumulation, which was also very low in *GRP1* plants (Figure 6e). These results suggested that while small amounts of virus are sufficient to induce leaf curling, much higher levels are required to cause leaf chlorosis. Arabidopsis plants infected with less virulent geminiviruses that often accumulate to lower levels also show leaf curling but no chlorosis (Aimone et al., 2021; Reyes et al., 2017).

The long and short protein isoforms corresponding to the Pla‐1 *GRP1* transgenes were detected in protein extracts but the long protein isoform produced from the Col‐0 transgene was not observed (Figure S6). This was unexpected because transcripts corresponding to all three transgenes were present at similar levels (Figure 6f). Thus, these results suggest that the accumulation of the Pla‐1 and Col‐0 protein orthologues is regulated differently at the level of translation or protein turnover. It is also possible that the endogenous Col‐0 AT1G31540 protein negatively affects the accumulation of the Col‐0 transgenic protein but not the Pla‐1 transgenic proteins. These possibilities can be addressed by future experiments that ask if a specific protein domain or amino acid sequence influences GRP1 accumulation and geminivirus resistance.

Many NLR receptor genes, including both CC and TIR types, have been reported to mediate resistance to infections by plant RNA viruses (Sett et al., 2022). To date, only two CC‐NLR genes, both identified in tomato, have been implicated in conferring resistance to geminivirus infection (Sharma et al., 2021; Yamaguchi et al., 2018). A common bean TIR‐NLR gene can deliver resistance to BDMV in a transient expression setting (Seo et al., 2007). In this study, we demonstrated that the TIR‐NLR gene from Arabidopsis Pla‐1, *GRP1*, can confer resistance to geminivirus infection in stably transformed plants, confirming the defense role of TIR‐NLR genes against geminivirus infection. Plant NLR proteins can be functionally categorized into sensor NLRs that recognize and interact with pathogen effectors and helper NLRs that transduce pathogen signals to downstream resistance pathways, with some singleton NLRs functioning in both ways (Contreras et al., 2023; Gong et al., 2023). The long isoform of the GRP1 protein has a TIR‐NBS‐LRR‐ID domain structure (Figure 6a; Figure S3). GRP1 is related to the well‐characterized RPP1 NLR that confers resistance to oomycete infection (Figure S7) (Botella et al., 1998; Contreras et al., 2023). The two NLRs have the same domain structures with a C‐terminal jelly roll/Ig‐like integrated domain (C‐JID) (Ma et al., 2020; Saucet et al., 2021). RPP1 (AT3G44480) is a sensor NLR that is activated by binding to the oomycete effector ATR1 (Botella et al., 1998; Ma et al., 2020) and triggers ETI by promoting dimerization of the EDS1 protein with PAD4 or SAG101 and activation of a network of helper NLRs, ultimately leading to the hypersensitive response (Contreras et al., 2023; Dongus & Parker, 2021). Because of its similarity to RPP1, GRP1 is also likely to act as a sensor NLR to confer geminivirus resistance.

The amino acid differences between the Pla‐1 and Col‐0 NLR alleles encoded by AT1G31540 occur almost entirely in their LRR and ID domains (Figure 6a; Figure S3a). These domains are likely involved in binding to an effector protein (Maruta et al., 2022). Failure of the short isoform of the Pla‐1 AT1G31540 NLR protein to confer resistance to Col‐0 suggested that the LRR and ID domains are necessary for productive effector binding (Figure 6; Figure S3). Thus, it is likely that the evolution of the Pla‐1 *AT1G31540* allele has resulted in an NLR with specific binding capacity for a CaLCuV effector protein that can elicit host defenses against geminivirus infection. The *AT1G31540* allele (*RAC1*) of the Arabidopsis Ksk‐1 accession confers resistance to oomycete infection (Borhan et al., 2004). The TIR and NBS domains of the Ksk‐1 and Pla‐1 AT1G31540 NLR proteins are also very conserved, while their LRR and ID domains are highly divergent, consistent with them recognizing different pathogen effectors (not shown). The *AT1G31540* genes in Pla‐1, Ksk‐1 and Col‐0 are not paired with a second NLR gene in the Arabidopsis genome, and Ksk‐1 *RAC1* oomycete resistance requires the presence of the *EDS1* gene (Borhan et al., 2004). These observations are consistent with the AT1G31540 TIR‐NLRs acting as pathogen sensors that function through EDS1 to activate a downstream network of helper NLRs and limit viral infection.

The identity of the CaLCuV effector protein that interacts with GRP1 is not yet known. TYLCV Rep and ToLCNDV C4 proteins are effectors that interact with the CC‐NLRs that confer geminivirus resistance in tomato (Sharma et al., 2021; Shen et al., 2020), but there is no information about the geminivirus effector proteins of TIR‐NLRs. Characterization of the CaLCuV protein(s) that activate GRP1 during ETI would lay the groundwork for using homologous proteins from other geminiviruses to screen for TIR‐NLRs that could confer viral resistance in other plant species. Alternatively, the LRR‐ID domains from GRP1 or other TIR‐type sensor NLRs that interact with geminivirus proteins could be fused to the TIR and NBS domains of closely related TIR‐NLRs to produce chimeric TIR‐NLRs that can activate plant host defense pathways in response to viral infection. The feasibility of using chimeric TIR‐type NLRs is supported by the presence of the downstream components of the ETI pathways involving EDS1 in many dicot species (Liu et al., 2023). A chimeric approach would target specific geminiviruses that cause agronomically important diseases and could also be readily adapted to emerging viruses. Reducing leaf chlorosis could help plants avoid the most adverse consequences of impaired photosynthesis due to leaf yellowing. Thus, *GRP1* has the potential to reduce crop losses associated with geminivirus diseases characterized by severe chlorosis.

## MATERIALS AND METHODS

### Pla‐1 genome sequencing

*Arabidopsis thaliana* ecotype Pla‐1 (ABRC stock CS28641, https://abrc.osu.edu/) genomic DNA from leaf tissues was used for sequencing library construction with the Illumina DNA Prep kit. The sequencing was carried out with Illumina's NextSeq 2000 System at the Genomic Sciences Laboratory, North Carolina State University. The 150‐bp paired‐end reads were aligned to the TAIR10 Col‐0 genome reference sequence using the BWA‐MEM tool. The alignments were visualized using IGV software (https://igv.org/).

### Genotyping and genetic mapping

Pla‐1 and Col‐0 plants and their crossing descendants were grown at 20°C with 8 h/16 h day‐night light cycles. The ecotypes were crossed by dusting Pla‐1 pistils with Col‐0 pollen, and the F_1_ plants were self‐pollinated to obtain F_2_ seeds for genotyping and genetic mapping. Approximately 5‐week‐old F_2_ plants were inoculated in the rosette center with a 1:1 mixture of *Agrobacterium* strains carrying CaLCuV A and B replicons (pNSB1090 and pNSB1091, respectively) (Egelkrout et al., 2002). Infection symptoms were scored at 28 dpi. The scores are: 1, no symptoms; 2, appearance of very mild leaf curling or chlorosis; 3, leaf curling or chlorosis well established in at least two leaves; 4, both leaf curling and chlorosis spread to wider areas in the rosette; and 5, severe leaf curling and chlorosis spread throughout the rosette. The day before inoculation, expanded leaves 4 and 5 counted from the rosette center were collected for genotyping. The leaves were ground in liquid nitrogen for 1 min using a Retsch MM301 grinder set at 30 rounds per minute. For total DNA extraction, the leaf powders (no more than 50 mg) were suspended in 350 μl of 100 mm Tris–HCl, pH 8.0, 20 mm EDTA, 1.4 m NaCl, 2% (w/v) cetyltrimethylammonium bromide and 1% (v/v) β‐mercaptoethanol and incubated at 65°C for 30 min (Allen et al., 2006). After centrifugation, the supernatant was extracted once with one volume of chloroform:isoamyl alcohol (24:1), and DNA was precipitated by isopropanol and washed with 70% (v/v) ethanol. Dried DNA pellets were dissolved in 100 μl water. The double‐stranded DNA (dsDNA) content was measured with the Quant‐i PicoGreen dsDNA Assay Kits (Invitrogen).

The KASP technology, which can differentially detect both types of homozygotes and the heterozygote at a SNP site, was used for genotyping the individual F_2_ plants (Semagn et al., 2014). The SNPs between Pla‐1 and Col‐0 were selected based on the alignment of the Pla‐1 genome sequence and the TAIR10 Col‐0 genome reference sequence. SNPs with intervals of 2.5 and 0.1 Mb were chosen for genome‐wide mapping and fine mapping, respectively (Tables S1 and S2). The DNA oligomers specific for each of the selected SNPs were designed and synthesized with KASP chemical tags by LGC Genomics. Genotyping was performed according to the procedures provided by LGC Genomics in a Bio‐Rad CFX Connect Real‐Time PCR Detection System, or by the service at LGC Genomics for high‐throughput reactions (https://biosearch‐cdn.azureedge.net/assetsv6/running‐KASP‐on‐BIO‐RAD‐CFX.pdf). The R software R/qtl (Broman & Sen, 2009) was used for mapping virus susceptibility/resistance QTLs using the nonparametric symptom scores 1–5. LOD values for a QTL at each marker SNP site were calculated and permutation tests with 1000 replicas were performed to obtain estimates of LOD thresholds for a significance level of *P* < 0.05. QTL regions were estimated according to a drop of 1.5 from the peak value or by using the 95% Bayes credible interval (Broman & Sen, 2009).

For QTL‐Seq, 200 ng of 50 randomly selected DNA samples from asymptomatic F_2_ plants (symptom score = 1) were pooled as a bulk DNA sample. Likewise, 50 DNA samples from CaLCuV‐infected plants with severe symptoms (symptom score = 5) were pooled to give a second bulk DNA sample. The pooled DNAs were concentrated by ethanol precipitation and dissolved in water. DNA libraries were made from the two DNA bulks with the Illumina DNA Prep kit and sequenced by Illumina's NextSeq 2000 System at the Genomic Sciences Laboratory, North Carolina State University. The 150‐bp paired‐end reads were aligned to the TAIR10 Col‐0 genome reference sequence using the BWA‐MEM tool. The Genome Analysis Toolkit suite of tools (GATK, https://gatk.broadinstitute.org/hc/en‐us) was used to call genome‐wide SNPs between the Pla‐1 and Col‐0 genomes. QTLs were identified by the R software QTLseqr (Mansfeld & Grumet, 2018; Takagi et al., 2013). Briefly, SNP indexes of the Pla‐1 sequence content in the bulk DNA from symptomless plants and the Col‐0 sequence content in the bulk DNA from severely infected plants were calculated for 300‐kb windows with 10‐kb steps along the Arabidopsis genome. QTL signals for Pla‐1 resistance were measured by the difference of the two indexes. Both 95% and 99% confidence intervals (CI) for the QTL signals were calculated using 10 000 rounds of simulated data (Mansfeld & Grumet, 2018).

### RNA‐Seq analysis

Pla‐1 and Col‐0 plants grown for 5 weeks at 20°C with 8 h/16 h day‐night light cycles were inoculated with CaLCuV at leaf number 5 counting from the rosette center by bombardment with gold particles coated with 2.5 μg per six plants each of the plasmids containing DNA‐A and DNA‐B replicons (pCPCbLCVA.003 and pCPCbLCVB.002, respectively) (Turnage et al., 2002). The empty vector pMON921 was used for mock infection. The inoculated leaves were collected and pooled at 4 dpi from four plants for one biological replicate. Leaves of untreated healthy plants at the same age were also collected. For every treatment, four biological replicates were prepared. Total RNAs were isolated using Qiagen's RNeasy Plant Mini Kit (Qiagen, Hilden, Germany) with the addition of DNase treatments following manufacturer's instructions. RNAs were eluted with water and quantified using the Qubit RNA BR Assay Kit (Invitrogen, Eugene, OR, USA). Poly(A) RNA was enriched and purified with the NEBNext Poly(A) mRNA Magnetic Isolation Module (New English Biolabs, Ipswich, MA, USA) from 500 ng of each of the total RNA samples and used for library preparation with the NEBNext Ultra II Directional RNA Library Prep Kit for Illumina according to the protocols provided by New England Biolabs. The resulting libraries were sequenced with Illumina's NextSeq 6000 Sequencing System at the Genomic Sciences Laboratory, North Carolina State University. The 150‐bp paired‐end reads were trimmed to remove adapter sequences using Cutadapt (https://cutadapt.readthedocs.io/) and aligned to the TAIR10 Arabidopsis Col‐0 (GenBank GCA_000001735) or Ler‐0 (GenBank GCA_900660825) genomes using HISAT2 (https://daehwankimlab.github.io/hisat2/). Gene counts were generated using htseq‐count (https://htseq.readthedocs.io/) according to the TAIR10 annotations. Quality control was performed using FastQC (https://www.bioinformatics.babraham.ac.uk/projects/fastqc/). Genes with less than 10 reads in three or fewer samples were filtered out. DGE analysis was performed with DESeq2 (https://bioconductor.org/packages/release/bioc/html/DESeq2.html). Wald tests were performed in DESeq2 using the DESeq commands and the *P* values attained were adjusted for multiple testing using the Benjamini and Hochberg method to identify differentially expressed genes. An adjusted *P* < 0.05 was considered significant DEG.

### Plasmid construction

The sequences of the primers (synthesized by Integrated DNA Technologies) used for cloning are listed in Table S5, and the plasmids used or generated in this study are listed in Table S6. All the restriction endonucleases and enzymes for molecular cloning were purchased from New England Biolabs. Final gene expression inserts were confirmed by Sanger sequencing.

The short *Bst*XI‐*Pme*I fragment of the binary plasmid vector pCAMBIA2300 was replaced with the short *Pac*I‐containing *Bst*XI‐*Pme*I fragment of pCAMBIA1300‐PacI, resulting in pNSB2159 to be used for insertion of gene expression constructs.

To clone the *UBQ1* promoter, a 675‐bp fragment immediately upstream of the coding region of the Arabidopsis *UBQ1* gene (*AT3G52590*) was amplified from Arabidopsis Col‐0 genomic DNA using the primer pair, ATUBQ1PROF (tagged with *Pst*I–*Pac*I–*Sac*II) and ATUBQ1PROR (tagged with *Sal*I‐*Spe*I), followed by digestion with *Pst*I and *Sal*I. A 303‐bp fragment containing the pea *rbcS‐E9* gene terminator was amplified from pMON921 using the primer pair, E9TERF (tagged with *Sal*I–*Cla*I) and E9TERR (tagged with *Eco*RI–*Pac*I–*Kpn*I), followed by digestion with *Sal*I and *Eco*RI. The promoter and terminator fragments and the vector plasmid pUC19 cut with *Pst*I and *Eco*RI were ligated together to generate pNSB2157, a Promoter^*UBQ1*^:Terminator^*rbcS‐E9*^ gene expression cassette.

To clone the *TCTP* promoter, a 303‐bp fragment immediately upstream of the coding region of the Arabidopsis *TCTP* gene (*AT3G16640*) was amplified from Arabidopsis Col‐0 genomic DNA using the primer pair, ATTCTPPROF (tagged with *Pst*I–*Pac*I) and ATTCTPPROR (tagged with *Sal*I–*Spe*I), followed by digestion with *Pst*I and *Sal*I. A 254‐bp fragment containing the *Agrobacterium NOS* gene terminator was amplified from pK7FWG2 using the primer pair, NOSTERF (tagged with *Sal*I–*Cla*I) and NOSTERR (tagged with *Eco*RI–*Pac*I–*Kpn*I–*Sac*II), followed by digestion with *Sal*I and *Eco*RI. The promoter and terminator fragments and the vector pUC19 cut with *Pst*I and *Eco*RI were ligated together to generate pNSB2158, a Promoter^*TCTP*^:Terminator^*NOS*^ gene expression cassette.

The Pla‐1 *AT1G31540.2* ORF was amplified from cDNA generated from Pla‐1 leaf mRNA with the forward primer, AT1G31540F, with an *Spe*I tag and the CAACA sequence immediately before the ATG start codon, and the reverse primer, AT1G31540HAR, with a *Sal*I tag and an in‐frame sequence encoding the HA peptide before the stop codon. The PCR product was digested with *Spe*I and *Sal*I and ligated to the backbone of *Spe*I/*Sal*I‐digested pNSB2157 to give pNSB2206, which contains the gene expression construct – Promoter^*UBQ1*^:ORF^*Pla‐AT1G31540.2*^‐HA:Terminator^*rbcS‐E9*^. The coding regions for the Col‐0 long isoform AT1G31540.2 and the Pla‐1 short isoform AT1G31540.1 were similarly amplified and cloned into pNSB2157 using the primer pairs, COL0AT1G31540F/AT1G31540HAR and AT1G31540F/PLA1AT1G31540.1R, respectively, from cDNAs derived from Col‐0 or Pla‐1 leaf mRNA to give the plasmids pNSB2272 and pNSB2273, respectively.

The Pla‐1 *AT1G32230.1 RCD1* ORF was amplified from cDNA derived from Pla‐1 leaf mRNA with the forward primer, AT1G32230F, with an *Spe*I tag and the CAACA sequence immediately before the ATG start codon, and the reverse primer, AT1G32230MYCR with an *Sal*I tag and an in‐frame sequence encoding the MYC peptide before the stop codon. The PCR product was digested with *Spe*I and *Sal*I and ligated to the backbone of *Spe*I/*Sal*I‐digested pNSB2158 to give pNSB2207, which contains the gene expression construct – Promoter^*TCTP*^:ORF^*Pla‐AT1G32230.1*^‐MYC‐Terminator^*NOS*^.

To express Pla‐1 *AT1G31540.2* and *AT1G32230.1* simultaneously from a single binary plasmid, the gene expression construct for *AT1G31540.2* in pNSB2206 was isolated by *Sac*II/*Kpn*I digestion and ligated with the backbone of *Sac*II/*Kpn*I‐digested pNSB2207 that contains the construct for *AT1G32230.1*, resulting in the dual expression cassette pNSB2208.

The gene expression constructs in pNSB2206, pNSB2207, pNSB2208, pNSB2272 and pNSB2273 were isolated by *Pac*I digestion and inserted into the *Pac*I site of pNSB2159 and the resulting plasmids, pNSB2209, pNSB2210, pNSB2211, pNSB2274 and pNSB2275, respectively. Clones with the expression cassettes and the kanamycin resistance gene *NPTII* in the vector in the same orientation were transformed into *Agrobacterium tumefaciens* strain GV3101:pMP90.

### Arabidopsis transformation, transgene expression evaluation, CaLCuV infection, and viral DNA measurement

Arabidopsis Col‐0 plants were transformed by the floral dip method (Clough & Bent, 1998) using the *Agrobacterium* strains containing the binary plasmids with the gene expression constructs and the *NPTII* kanamycin resistance gene as the selection marker. Homozygous T_3_ lines that contain a single locus of T‐DNA insertion were selected and characterized for transgene expression levels and in CaLCuV infection assays.

Transgenic and wild‐type Col‐0 plants were grown at 20°C with 8 h/16 h day‐night light cycles. Approximately 5‐ to 6‐week‐old plants were used to assess transgene expression. The four youngest leaves and shoot apical meristem tissues at the rosette center were collected for RNA extraction as described above for RNA‐Seq. To generate cDNAs, total RNA (2.5 μg) in 10 μl of water was incubated with 100 units of M‐MuLV reverse transcriptase (New England Biolabs) in the presence of 50 ng μl^−1^ oligo (dT)_18_ (New England Biolabs) at 42°C for 1 h, followed by dilution into 250 μl of water. End‐product PCR was carried out with 2 μl cDNA, 0.2 μm each of the primers and 10 μl Hot Start *Taq* 2X Master Mix (New England Biolabs) in a total volume of 20 μl. After an initial incubation at 95°C for 30 sec, 35 thermal cycles of 95°C for 30 sec, 60°C for 30 sec and 68°C for 30 sec were applied. The primers used for RNA transcribed from the *AT1G31540.2* transgene were PLA11G31540RTF1 and HARTR and the primers for RNA synthesized from the *AT1G32230.1* transgene were PLA11G32230RTF1 and MYCRTR. Quantitative PCR was performed in 20 μl reactions containing 5 μl of diluted cDNA, 5 μl of 1 μm each of the forward and reverse primers and 10 μl of PowerUp SYBR Green Master Mix (Applied Biosystems, Vilnius, Lithuania) that included the thermal DNA polymerase and dNTPs. After initial incubation at 50°C for 2 min and 95°C for 2 min, the qPCR reactions were run with 40 cycles of 95°C for 15 sec, 60°C for 15 sec, and 72°C for 1 min. Arabidopsis *AP2M* (*AT5G46630*) RNA was used as the internal reference (Czechowski et al., 2005). The primer pairs were AT1G31540RTF3/HARTR for the Pla‐1 and Col‐0 *AT1G31540.2* transgenes, AT1G31540SEQF4/HARTR for the Pla‐1 *AT1G31540.1* transgene, and AP2MRTF/AP2MRTR for the *AP2M* reference (Table S5). Copy numbers of the transgene transcripts for the long and short isoforms of the *AT1G31540* gene products were determined according to the standard curves derived from the plasmids pNSB2206 and pNSB2273, respectively, and their log_2_ values were normalized against the Ct values from the reactions for the *AP2M* reference gene. The resulting data were arbitrarily compared to the line PlaN‐2C4, and finally, the relative gene expression levels were displayed in linear scale.

Equivalent plant materials were collected for soluble total protein extraction. Frozen leaf tissue pooled from three plants was ground in a Retsch MM301 grinder set at 30 rounds per minute for 1 min. The powders were incubated at 22°C for 10 min in two volumes of an extraction buffer containing 25 mm Tris–HCl, pH 7.5, 140 mm NaCl, 2.7 mm KCl, 1 mm EDTA, 0.2% Triton X‐100, 5% (v/v) glycerol, 50 μm MG132 and 1 × Thermo Scientific's Halt Protease Inhibitor Cocktail. After centrifugation to remove cell debris, 2.5 mg of extracted protein was adjusted to a 300‐μl final volume with the extraction buffer and applied to antibody‐based Pierce Anti‐HA Magnetic Beads (Thermo Scientific) for immunoprecipitation according to the manufacturer's instructions. The bound proteins were eluted in 100 μl of SDS‐PAGE sample buffer without reducing reagent by boiling for 5 min, and DTT was added to the supernatants to a 50‐mm final concentration. Eluted proteins (50 μl) were applied to a 4–20% gradient gel for SDS‐PAGE and transferred to a PVDF membrane. The membrane was incubated with 2 μg ml^−1^ of mouse anti‐HA tag monoclonal antibody (Sigma Aldrich) and subsequently with a 1:2000 dilution of HRP‐conjugated sheep anti‐mouse IgG antibodies (Millipore Sigma). After a 5‐min incubation with the SuperSignal West Pico PLUS Chemiluminescent Substrate (Thermo Scientific), immunoblot images were recorded with a Syngene G:BOX Chemi HR16 Gel Imaging System. Aliquots of 25 μg total proteins were subjected to SDS‐PAGE and Coomassie Brilliant Blue staining to control for immunoprecipitation protein input.

For CaLCuV infection assays, Arabidopsis plant rosette center was inoculated with *Agrobacterium* lines containing viral DNA replicons or bombarded with 0.1 μg per six plants of each of the plasmids containing DNA‐A and DNA‐B replicons as described above. The *Agrobacterium* strain or the plasmid containing viral DNA‐B replicons was used for mock infection. Symptoms of leaf curling and leaf chlorosis were scored separately on 1–5 scales: 1, no symptoms; 2, very mild symptoms often indistinguishable from mock‐infected plants; 3, symptoms firmly established in at least two leaves; 4, symptoms spread to more leaves; and 5, almost the entire plant showing very severe symptoms. The young leaves and apical meristem tissues were collected from infected plants at 8 and 14 dpi to determine the copy number of CaLCuV DNA‐A. Total DNA was extracted as described above for genotyping but with 10 mm DTT replacing β‐mercaptoethanol and with the addition of 1% PVP in the extraction buffer. A phenol:chloroform:isoamyl alcohol (25:24:1) extraction step was also added before the chloroform:isoamyl alcohol extraction. After treatment with 10 μg ml^−1^ RNase A in water at 37°C for 30 min, the DNA samples were extracted with phenol:chloroform:isoamyl alcohol and then with chloroform:isoamyl alcohol, reprecipitated with ethanol and dissolved in 500 μl of water. The dsDNA content was measured with the Qubit dsDNA HS Assay Kit (Invitrogen). A plasmid containing the CaLCuV DNA‐A replicon was used to generate qPCR standard curves to quantify DNA‐A copy number. The qPCR reactions contained 5 μl of diluted DNA, 5 μl of 1 μm each of the primers, CaLCuV1990F and CaLCuV1990R (Table S5) (Rajabu et al., 2018), and 10 μl of PowerUp SYBR Green Master Mix (Applied Biosystems). After initial incubation at 50°C for 2 min and 95°C for 2 min, the qPCR reactions were run with 40 cycles of 95°C for 15 sec, 50°C for 15 sec and 72°C for 30 sec. Viral copy number was normalized to total dsDNA content per sample.

## AUTHOR CONTRIBUTIONS

LH‐B, JTA‐I and JN acquired the funding. LH‐B, JTA‐I, WS, and MIR conceived and designed the experiments. WS, MIR, DD, LM‐Y performed the experiments. WS, RR, and EW did the data analyses. WS, LH‐B and JTA‐I wrote the manuscript with contributions from RR, EW and LM‐Y. All the authors read and approved the manuscript.

## CONFLICT OF INTEREST

Part of the data presented in this manuscript has been used for patent applications to the United States Patent and Trademark Office (Patent No. 12305184). The authors declare no additional conflict of interest.

## Supporting information


**Figure S1.** Detailed data for the analyses of the contribution of *GRP3* to the Pla‐1 resistance against CaLCuV infection shown in Figure 3.
**Figure S2.** Distribution of gene expression and protein polymorphism of the protein coding genes in the Pla‐1 *GRP1* locus.
**Figure S3.** Alignment of the amino acid sequences of the Pla‐1 and Col‐0 AT1G31540 NLR proteins.
**Figure S4.** Transgenes in Arabidopsis Col‐0 lines used to access candidate *GRP1* genes are expressed in plants.
**Figure S5.** Resistance conferred by Pla‐1 AT1G31540.2 NLR is independent of the mode of CaLCuV inoculation.
**Figure S6.** Protein production of various forms of the C‐terminally HA‐tagged AT1G31540 NLR transgenes in transgenic Arabidopsis Col‐0 plants.
**Figure S7.** A phylogenetic tree of the Arabidopsis TIR‐NLR proteins showing the evolutionary position of GRP1 TIR‐NLR.

**Table S1.** SNP markers used for fine‐mapping of *GRP1*.
**Table S2.** SNP markers used for mapping of *GRPs* in the whole genome of Pla‐1.
**Table S3.** Gene models in the *GRP1* locus downloaded from TAIR10.
**Table S4.** Expressed protein coding genes in the mapped *GRP1* locus.
**Table S5.** Primers used in this study.
**Table S6.** Plasmids used or generated in this study.

## Data Availability

The data supporting the findings are available within the article, in supporting information, or by request to the corresponding authors. The sequence reads generated from the Arabidopsis Pla‐1 genome and RNA‐Seq experiments were deposited in the National Center for Biotechnology Information (NCBI) Sequence Read Archive with the accession number PRJNA1138721. The Arabidopsis Pla‐1 mRNA sequences for the long (AT1G31540.2) and short (AT1G31540.1) isoforms of TIR‐NLR proteins were deposited in NCBI's GenBank database with the accession numbers PV383299 and PV383300, respectively.
