# Supplementary material for: A TIR‐NLR gene from Arabidopsis Pla‐1 confers resistance to geminivirus infection
Source: Plant J. 2025 Dec 6;124(5):e70628. doi: 10.1111/tpj.70628 (PMC12680929; doi:10.1111/tpj.70628)
Supplement: Supplementary file 1 — Figure S1. Detailed data for the analyses of the contribution of GRP3 to the Pla‐1 resistance against CaLCuV infection shown in Figure 3. Figure S2. Distribution of gene expression and protein polymorphism of the protein coding genes in the Pla‐1 GRP1 locus. Figure S3. Alignment of the amino acid sequences of the Pla‐1 and Col‐0 AT1G31540 NLR proteins. Figure S4. Transgenes in Arabidopsis Col‐0 lines used to access candidate GRP1 genes are expressed in plants. Figure S5. Resistance conferred by Pla‐1 AT1G31540.2 NLR is independent of the mode of CaLCuV inoculation. Figure S6. Protein production of various forms of the C‐terminally HA‐tagged AT1G31540 NLR transgenes in transgenic Arabidopsis Col‐0 plants. Figure S7. A phylogenetic tree of the Arabidopsis TIR‐NLR proteins showing the evolutionary position of GRP1 TIR‐NLR. [file TPJ-124-0-s002.pdf]

(a)

Population	*GRP3* genotype	Average symptom score	Number of plants	SE
Wild-type Col-0	Col-0/Col-0	4.63	138	0.05
Pla-1 x Col-0 F_2	Col-0/Col-0	3.21	561	0.06
	Pla-1/Col-0	3.05	1118	0.04
	Pla-1/Pla-1	2.71	475	0.07
Wild-type Pla-1	Pla-1/Pla-1	1.17	125	0.05

(b)

Population	*GRP1* genotype	*GRP3* genotype	Average symptom score	Number of plants	SE
Pla-1 x Col-0 F_2	Col-0/Col-0	Col-0/Col-0	4.61	72	0.09
		Pla-1/Col-0	4.56	116	0.08
		Pla-1/Pla-1	4.46	46	0.15
	Pla-1/Col-0	Col-0/Col-0	3.37	158	0.10
		Pla-1/Col-0	3.15	373	0.07
		Pla-1/Pla-1	2.70	158	0.11
	Pla-1/Pla-1	Col-0/Col-0	2.15	106	0.11
		Pla-1/Col-0	1.91	194	0.08
		Pla-1/Pla-1	1.87	110	0.11

Figure S1. Detailed data for the analyses of the contribution of *GRP3* to the Pla-1 resistance against CaLCuV infection shown in Figure 3.
(a) Data for Figure 3c. SE, standard error.
(b) Data for Figure 3d. SE, standard error.

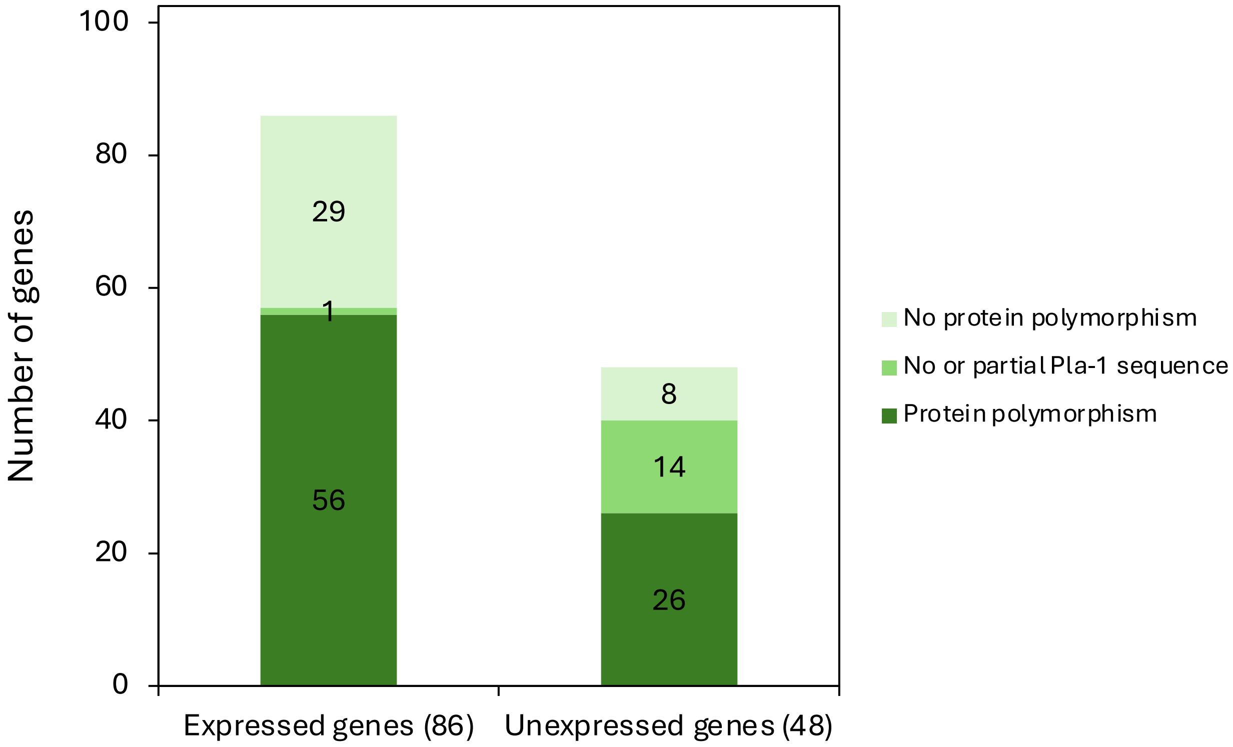

Figure S2. Distribution of gene expression and protein polymorphism of the protein coding genes in the Pla-1 GRP1 locus.

RNA-Seq data was generated from Pla-1 and Col-0 plants at 4 days post treatment of leaf 5. A protein coding gene in the Pla-1 GRP1 locus was called as expressed if sequencing reads mapped to the gene using the Col-0 TAIR10 as reference genome for at least one of the treatments (untreated, mock-inoculated, or CaLCuV-inoculated) of either ecotype. Protein polymorphisms were identified by comparing the deduced amino acid sequences of the Pla-1 gene products with the Col-0 sequences. In some Col-0 gene loci, no or only partial Pla-1 genomic sequences aligned to the Col-0 sequences.

(a)

```
Pla-1   MASSSSSHNWLYDVFLSFRGEDVRVTFRSHFLKELDRKLITAFRDNEIERSHSLWPDLEQ     60
Col-0   MASSSSSHNWLYDVFLSFRGEDVRVTFRSHFLKELDRKLITAFRDNEIERSHSLWPDLEQ     60
                   <---------------- TIR ---------------------------

Pla-1   AIKESRIAVVVFSINYASSSWCLNELLEIVNCNDKIVIPVFYHVDPSQVRHQIGDFGKIF    120
Col-0   AIKESRIAVVVFSKNYASSSWCLNELLEIVNCNDKIVIPVFYHVDPSQVRHQIGDFGKIF    120
        --------------------------- TIR ---------------------------

Pla-1   ENTCKRQTDEEVKNQWKKALTLVANMLGFDSAKWNDEAKMIEEIANDVLGKLLLTTPKDS    180
Col-0   ENTCKRQTDEEVKNQWKKALTLVANMLGFDSAKWNDEAKMIEEIANDVLGKLLLTTPKDS    180
        --------------------------- TIR ----------------------->

Pla-1   EELVGIEDHIAEMSLLLQLESEEVRMVGISGSSGIGKTTIARALFKRLSRHFQGSTFIDR    240
Col-0   EELVGIEDHIAEMSLLLQLESKEVRMVGISGSSGIGKTTIARALFKRLSRHFQGSTFIDR    240
            <---------------------- NBS ---------------------------

Pla-1   AFVSYSRNIYSGANPDDPNMKLQLQGHFLSEILGKKDIKIDDPAALEERLKHQKVLIIID    300
Col-0   AFVSYSRNIYSGANPDDPNMKLQLQGHFLSEILGKKDIKIDDPAALEERLKHQKVLIIID    300
        --------------------------- NBS ---------------------------

Pla-1   DLDDIMVLDTLVGQTQWFGYGSRIIVVTNDKHFLIAHGIDHIYEVSFPTDVHACQMLCQS    360
Col-0   DLDDIMVLDTLVGQTQWFGYGSRIIVVTNDKHFLIAHGIDHIYEVSFPTDVHACQMLCQS    360
        --------------------------- NBS ---------------------------

Pla-1   AFKQNYAPKGFEDLVVDVVRHAGNFPLGLNLLGKYLRRRDMEYWMDMLPRLENSLRIDGK    420
Col-0   AFKQNYAPKGFEDLVVDVVRHAGNFPLGLNLLGKYLRRRDMEYWMDMLPRLENSLRIDGK    420
        --------------------------- NBS ---------------------------

Pla-1   IEKILRISYDGLESEDQEIFRHIACLFNHMEVTTIKSLLADSDVSFALENLADKSLIHVR    480
Col-0   IEKILRISYDGLESEDQEIFRHIACLFNHMEVTTIKSLLADSDVSFALENLADKSLIHVR    480
        --------------------------- NBS ---------------------------

Pla-1   QGYVVMHRSLQEMGRKIVRIQSIDKPGEREFLVDPNDIHDILNACTGTQKVLGISLDIRN    540
Col-0   QGYVVMHRSLQEMGRKIVRIQSIDKPGEREFLVDPNDIHDILNACTGTQKVLGISLDIRN    540
        -------------------->    <---------------------------------

Pla-1   IRELDVHERAFKGMSNLRFLEIKNFGLKEDGLHLPPSFDYLPRTLKLLCWPKFPMRCMPF    600
Col-0   IRELDVHERAFKGMSNLRFLEIKNFGLKEDGLHLPPSFDYLPRTLKLLCWSKFPMRCMPF    600
        -------------------------- LRR I --------------------------

Pla-1   GFRPENLVKLEMQCSKLHKLWEGVVPLACLKEMDLRVSLKLKVIPDLSEATNLEIILNLSF    660
Col-0   GFRPENLVKLEMQYSKLHKLWEGVAPLTCLKEMDLHGSSNLKVIPDLSEATNLEIILNLKF    660
        -------------------------- LRR I --------------------------

Pla-1   CESLVELPSSIRNLNKLLNLDMFYCKSLKILPTGFNLKSLDRLHLDHCSKLKTFPKFSTN    720
Col-0   CESLVELPSSIRNLNKLLNLDMLNCKSLKILPTGFNLKSLDRLNLYHCSKLKTFPKFSTN    720
        -------------------------- LRR I --------------------------

Pla-1   ISVLSLNLTNIEDFPSNLHLQNLVEFSISKDESDEKQWEEEKPLTPFLAMMLSPTLTILH    780
Col-0   ISVLNLNLTNIEDFPSNLHLENLVEFRISKEESDEKQWEEEKPLTPFLAMMLSPTLTSLH    780
        ----------------------------->          <------------------

Pla-1   LCDMPSLVELPSSFQNLNQLKELIIINCINLETLPTGINLQSLYYLSFRGCSQLRSFPEI    840
Col-0   LENLPSLVELTSSFQNLNQLKDLIIINCINLETLPTGINLQSLDYLCFSGCSQLRSFPEI    840
        -------------------------- LRR II -------------------------

Pla-1   STNISVLYLDETAIEEVPWWIEKFSNLTELRMDRCSRLKCVFLHISKLKHLQEALFRNCG    900
Col-0   STNISVLYLDETAIEEVPWWIEKFSNLTELSMNSCSRLKCVFLHMSKLKHLKEALFRNCG    900
        -------------------------- LRR II -------------------------

Pla-1   TLTRVELSGYPSGMEVMKADNIDTASSSLPKVVLSFLDCFNLDPETVLHHQESIIFNYML    960
Col-0   TLTRVELSGYPSGMEVMKADNIDTASSSLPKVVLSFLDCFNLDPETVLHHQESIIFNYML    960
        -------------------------- LRR II -------------------------

Pla-1   FTGKGEVPSYFTYRTTGSSSLTIPLLHVHLSQPFFRFRIGALVTIVNTEEPVELEVKCEF   1020
Col-0   FTGKEEVPSYFTYRTTGSSSLTIPILHVHLSQPFFRFRIGALVT--NKEEPVELEVKCEF   1018
        ->          <---------- ID (C-JID) ------------------------

Pla-1   KDRFGNNFDYDIYFEVYNQNYCVEDDYIIAILDCRIPLNEDNAALAQPNYYDHVDIKIEQ   1080
Col-0   KDRFGNNFDYDIYFEVNKDRYYGEDCYNIAILDCRIPLNEDNAALAQRNYYDHVDIKIEQ   1078
        ------------------------ ID (C-JID) ------------------------

Pla-1   LEEEERYGDIEQWGIRLLEDCSSAETRLDN-SNSTLPHVSEAEEGNIGYTPLQGLVNEIE   1139
Col-0   LD--KRYSDIEQWGIRLLEDCSSAETRLDNYSNSTLPHVSEAEEGNKGYTPLQGLVNEIE   1136
        ----------------->

Pla-1   HSEEPGDINVETERSTKRMRLYHFI                                      1164
Col-0   HSEEPGDINVETERSTKRMRLYHFI                                      1161
```

(b)

```
Pla-1   ------------------------------------------VSSSKLNIISKLFS       776
Col-0   ------------------------------------------VSSSTLNIISKLFP       776
```

Figure S3. Alignment of the amino acid sequences of the Pla-1 and Col-0 AT1G31540 NLR proteins.
(a) Alignment of the amino acid sequences of the long isoform AT1G31540.2 NLR proteins. Polymorphisms between the two proteins are highlighted in yellow. Sequences in black are identical between the long and short isoforms within an ecotype and sequences in green at the C-terminus are specific to the long isoforms. The domain boundaries, indicated by underlines and arrows, are determined by analyzing the Pla-1 protein sequence in the databases of PROSITE Profile, and NCBI-CDD and Pfam and consulting the AlphaFold Protein Structure Database for the 3D structure model of the Col-0 protein.
(b) Alignment of the C-terminal sequences (in red) specific to the short isoform AT1G31540.1 NLR proteins. Polymorphisms between the two proteins are highlighted in yellow.

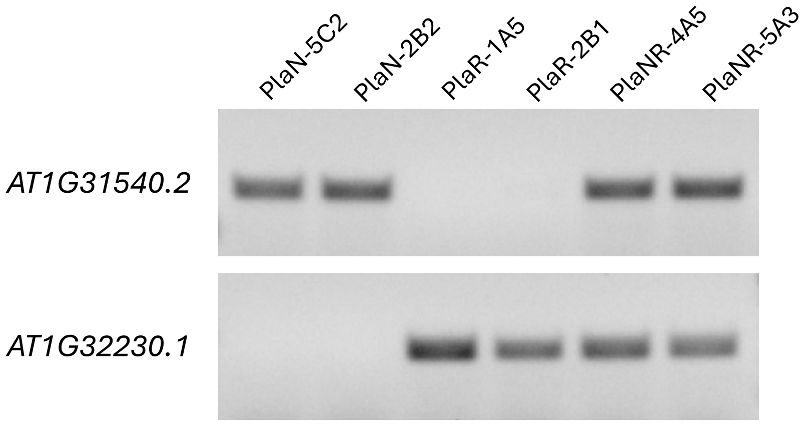

Figure S4. Transgenes in Arabidopsis Col-0 lines used to access candidate *GRP1* genes are expressed in plants.

Complementary DNAs were derived from plants of selected T_3 generation transgenic lines expressing Pla-1 *AT1G31540.2* (PlaN), *AT1G32230.1* (PlaR), or both (PlaNR) and subjected to PCR with primers specific to the transgenes. PCR end-products were resolved by agarose gel electrophoresis.

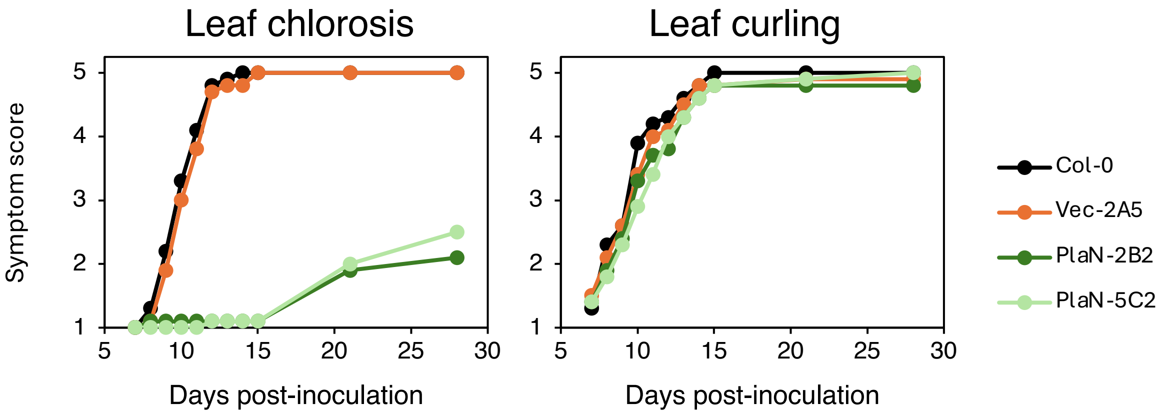

Figure S5. Resistance conferred by Pla-1 AT1G31540.2 NLR is independent of the mode of CaLCuV inoculation.

Plants were inoculated using a microsprayer and gold particles coated with CaLCuV DNA-A and DNA-B replicon plasmids. Plots of leaf chlorosis and curling, which were scored separately at multiple timepoints post-inoculation, are shown. The analysis compared wild-type Col-0 plants with plants from 1 T_3 Vec line (empty vector control) and 2 T_3 PlaN lines (Pla-1 *AT1G31540.2* expression cassette) (n = 6).

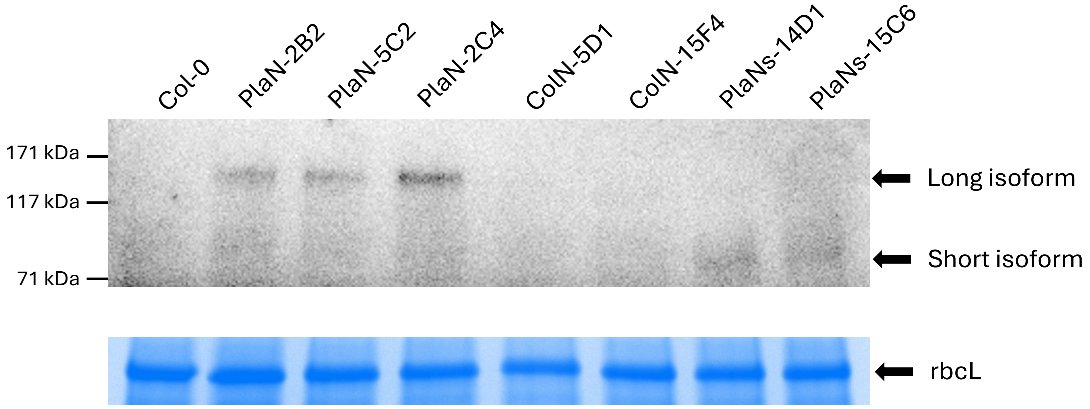

Figure S6. Protein production of various forms of the C-terminally HA-tagged AT1G31540 NLR transgenes in transgenic Arabidopsis Col-0 plants.
Soluble proteins were extracted from uninfected Arabidopsis plants of transgenic lines expressing the long isoform of Pla-1 gene (PlaN; AT1G31540.2), the short isoform of Pla-1 gene (PlaNs; AT1G31540.1/.3), or the long isoform of Col-0 gene (ColN; AT1G31540.2). HA-tagged proteins were immunoprecipitated by immobilized anti-HA antibodies and detected by immunoblotting with anti-HA antibodies (upper panel). SDS-PAGE and subsequent Coomassie Brilliant Blue staining of the protein extracts was performed for protein input control for immunoprecipitation (lower panel). The bands corresponding to the large subunit of Rubisco (rbsL) are shown.

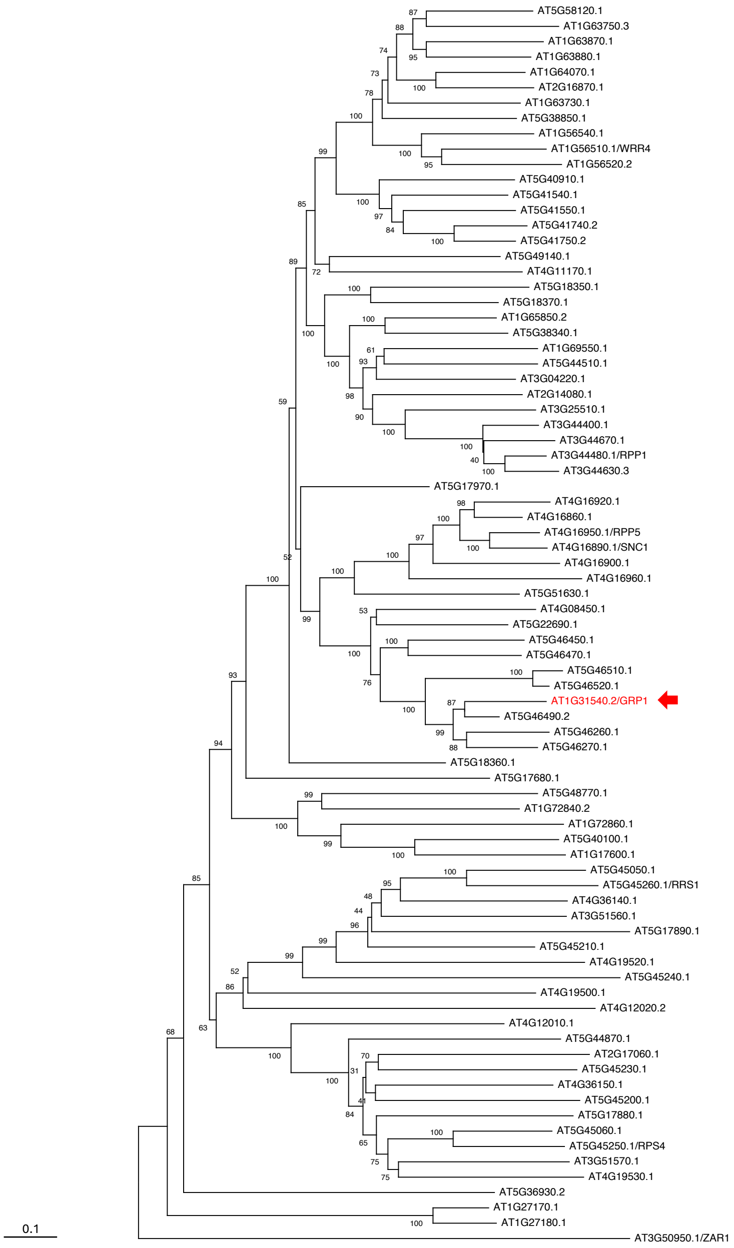

Figure S7. A phylogenetic Tree of the Arabidopsis TIR-NLR proteins showing the evolutionary position of GRP1 TIR-NLR.
Arabidopsis Col-0 TIR-NLR proteins were retrieved from the TAIR10 database. Only full-length proteins that contain a minimal TIR-NBS-LRR domain structure were included in building the neighbor-joining phylogenetic tree using the MEGA X software [Kumar et al. (2018) MEGA X: Molecular evolutionary genetics analysis across computing platforms, Molecular Biology and Evolution 35, 1547-1549]. Arabidopsis CC-NLR protein ZAR1 was used as the outgroup. Percentages of support from bootstrap tests of 1000 replicas are shown. The scale bar indicates 0.1 amino acid substitutions per site. The Col-0 allele of Pla-1 GRP1 (AT1G31540.2) is shown in red. Names for some of the characterized TIR-NLR proteins are included.
